# VPS28 regulates triglyceride synthesis via ubiquitination in bovine mammary epithelial cells

**DOI:** 10.1038/s41598-024-82774-0

**Published:** 2024-12-28

**Authors:** Lily Liu, Jinhai Wang, Xianrui Zheng, Qin Zhang

**Affiliations:** 1 College of Biological and Food Engineering, Southwest Forestry University, Kunming, 650224 China; 2 The Roslin Institute, University of Edinburgh, Edinburgh, EH25 9RG UK; 3 College of Animal Science and Technology, Anhui Agricultural University, Hefei, 230036 China; 4 College of Animal Science and Technology, Shandong Agricultural University, Tai’an, 271018 China

**Keywords:** VPS28, Ubiquitination, Triglyceride, MAC-T cells, Mouse model, Genetics, Molecular biology

## Abstract

VPS28 (vacuolar protein sorting 28) is a subunit of the endosomal sorting complexes required for transport (ESCRTs) and is involved in ubiquitination. Ubiquitination is a critical system for protein degradation in eukaryotes. Considering the recent findings on the role of ubiquitination in the regulation of lipid metabolism, we hypothesized that VPS28 might affect the expression of genes involved in milk fat synthesis. To test this hypothesis, we modulated VPS28 expression in the bovine mammary epithelial cell line (MAC-T) and measured the effects on triglyceride (TG) synthesis using lentivirus-mediated techniques. The results showed that VPS28 knockdown significantly upregulated the levels of the fatty acid transporter CD36 molecule (CD36) and adipose differentiation-related protein (ADFP), leading to increased TG and fatty acid production, along with elevated ubiquitin (UB) levels, while reducing proteasome activity. In contrast, VPS28 overexpression increased CD36 levels while not significantly affecting ADFP or TG levels, with a trend toward reduced lipid droplets and increased UB expression and proteasome activity. In addition, inhibition of the ubiquitin-proteasome system and the endosomal-lysosomal pathway using epoxomicin and chloroquine, respectively, further increased CD36, ADFP, and TG levels, thereby enhancing cell viability. These *in* *vitro* findings were validated in vivo in a mouse model, where VPS28 knockdown increased mammary CD36, ADFP, UB expression, TG content, and lipid droplets without pathological changes in mammary tissue or blood TG alterations. These results confirm the pivotal role of VPS28 in regulating TG synthesis via the ubiquitination pathway, offering novel insights into the molecular mechanisms of milk fat production in a bovine cell model.

## Introduction

Milk is a nutrient-rich food, with milk fat being a key indicator of quality, composed primarily of triglycerides (TGs). TGs serve as a source of energy storage and as a means of delivering nutrients, improving taste, and maintaining the structural stability of the product. During early lactation in dairy cows, triglyceride synthesis is primarily dependent on de novo fatty acid (FA) synthesis^1^. During peak lactation, long-chain FAs absorbed from the blood are predominantly utilized for triglyceride synthesis^1^. Consequently, investigations into the processes, factors, and mechanisms involved in milk fat production in mammary epithelial cells (MECs) are of significant importance for improving milk production and quality.

Milk fat synthesis in MECs involves multiple steps, including de novo FA synthesis, FA transport, TG synthesis, and lipid droplet formation and secretion. A number of molecules are involved in the regulation of this process. For instance, the ubiquitination pathway represents a fundamental eukaryotic cellular mechanism in regulating protein degradation^2^. This ubiquitination pathway can directly regulate the transport of FAs and formation of lipid droplet via CD36 molecule (CD36)^3–5^ and the adipose differentiation-related protein (ADFP)^6–8^. In bovine MECs, CD36 is a key lipid receptor involved in the binding and internalization of various lipids^9,10^. While ADFP is mainly located on the surface of lipid droplets, where it is involved in fatty acid synthesis, as well as in the transport and exchange of lipids^11^.

Our previous studies have identified a C-58T mutation in the VPS28 gene, that was associated with reduced expression of VPS28 and significantly linked to higher milk fat percentage in Holstein dairy cows, and there was tissue-specific expression of VPS28 in the bovine mammary gland, suggesting that VPS28 may play a role in milk fat synthesis^12,13^. VPS28 is an integral component of the endosomal sorting complexes required for transport (ESCRT) complex^14,15^, which is essential for endocytosis^16,17^, protein sorting^18,19^, and membrane protein degradation^20,21^ through the endosomal-lysosomal pathway (ELP). Previous studies have shown that ESCRT complexes could affect lipid droplet dynamics by regulating vacuole functionality, in particular, ESCRT loss could lead to the accumulation of lipid droplet-derived diacylglycerol and inhibit its conversion to phosphatidic acid and membrane lipids^22^. In addition, VPS28 also influences the function of the ubiquitin-proteasome system (UPS) to regulate the ubiquitin-mediated degradation and helps maintain the balance of receptor-mediated signaling pathways^23–25^. Emerging evidence indicates that ubiquitination is a hub and driver of cellular lipid metabolism through regulating the levels of transporters or some membrane proteins^26–31^. For example, the UPS could promote the ubiquitination of the ligand-binding domain of peroxisome proliferator-activated receptors (PPARs), and then act as a negative feedback loop that limits the transcriptional activity of PPARs, resulting in the alteration of FA metabolism^38^. Few studies also indicate that the UPS controls the degradation of sterol regulatory element-binding proteins to affect the lipid metabolism^32,33^.

With respect to VPS28, the deletion of VPS28 was observed to result in a reduction in the expression of cell surface proteins in the mouse^34^. In yeast, VPS28 knockouts fail to deliver ubiquitinated cargo to the vacuole^15^. In humans, deficient VPS28 resulted in moderate defects in both biosynthetic and endocytic trafficking to the vacuole^35^. Nevertheless, further investigation is required to fully elucidate the direct impact of VPS28 on TG and milk fat synthesis in bovine cells.

In this sense, the objective of this study was to explore the regulatory mechanisms of VPS28 in milk fat synthesis in vitro using bovine mammary epithelial cells (MAC-T) and its function in lipid metabolism in vivo using a mouse model. The anticipated outcomes will contribute to our understanding of the molecular mechanisms of milk fat production.

## Results

### Effects of VPS28 alteration and inhibitors on cell viability in MAC-T cells

**Fig. 1:**
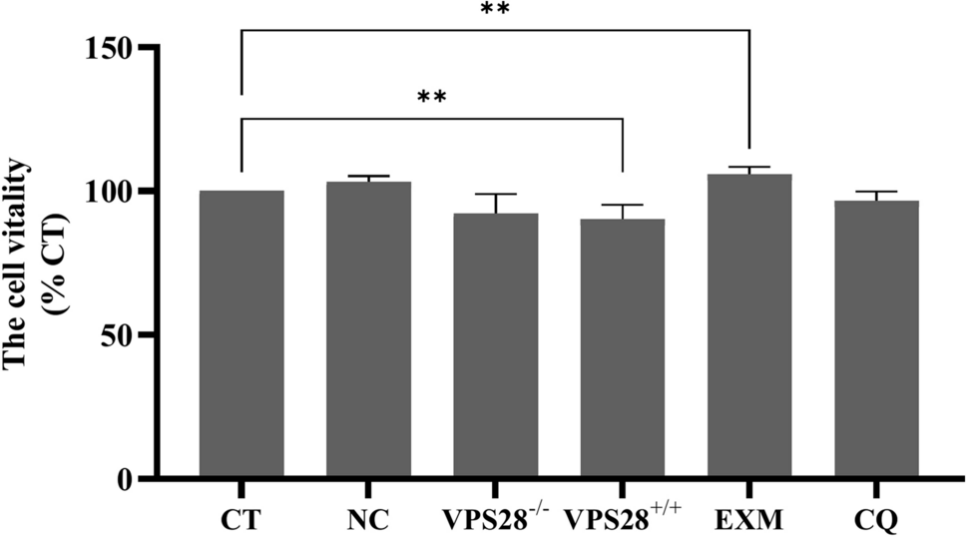
The cell vitality in each group. Treatments were replicated 4 times. The values are means ± SEM. VPS28^−/−^: VPS28 knockdown. VPS28^+/+^: VPS28 overexpression. **Denote significant differences (*P* < 0.01, respectively).

The cytotoxicity assay results revealed that both VPS28 knockdown and the application of the lysosome inhibitor chloroquine (CQ) had a negligible impact on the viability of MAC-T cells (*P* > 0.05). In contrast, overexpression of VPS28 was found to markedly reduce cell viability in comparison with the control group, as evidenced by a significant decrease (*P* < 0.01). Furthermore, exposure to the proteasome inhibitor epoxomicin (EXM) resulted in a significant increase in the viability of MAC-T cells (*P* < 0.01) (Fig. 1).

### VPS28 knockdown promotes milk fat synthesis in MAC-T cells

**Fig. 2:**
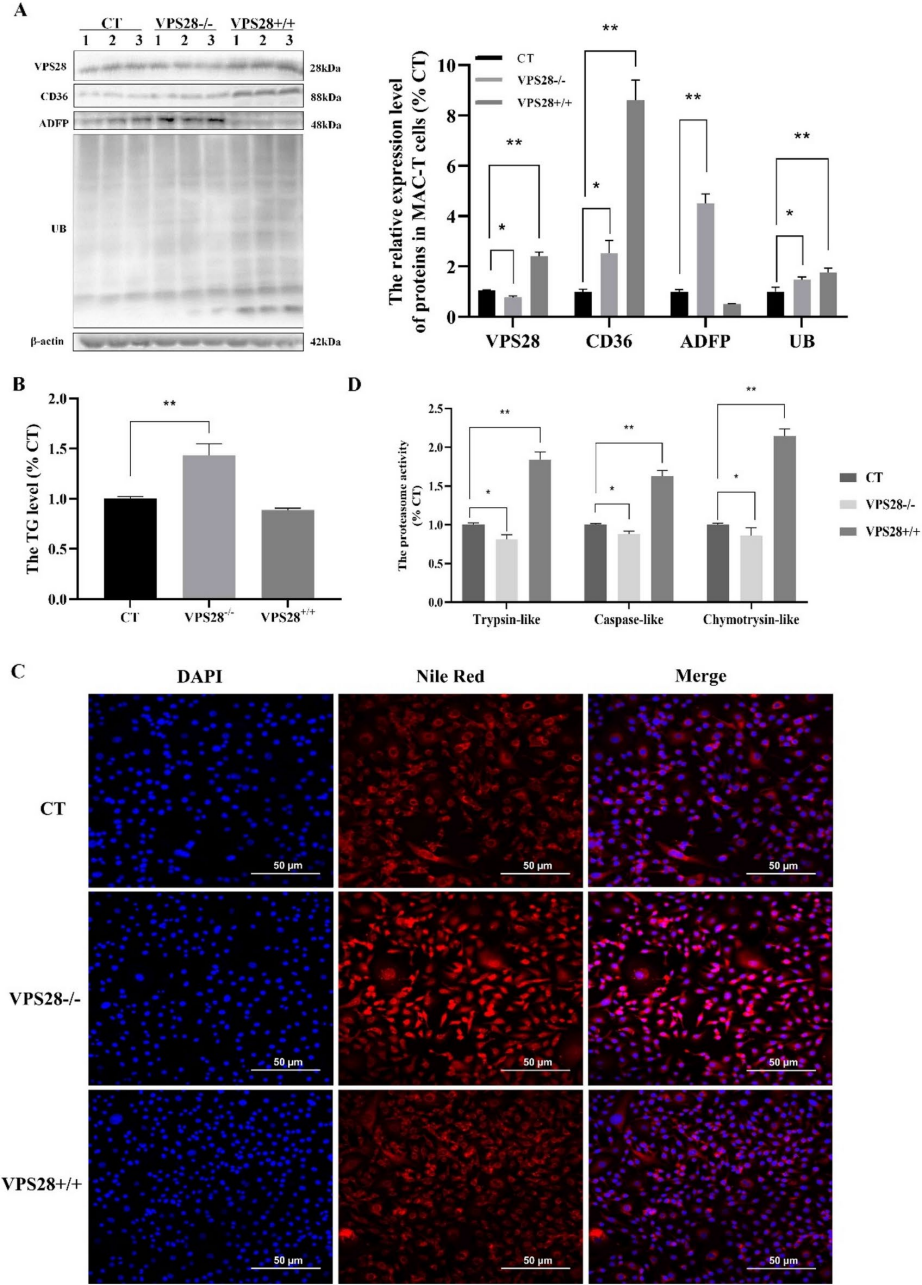
The effects of VPS28 on the MAC-T cells. (**A**) The relative protein expression levels. (**B**) The relative TG content. (**C**) The Nile Red staining. (**D**) The three activities of proteasome. CT: control. VPS28^−/−^: VPS28 knockdown. VPS28^+/+^: VPS28 overexpression. All treatments were independently replicated three times. Data are presented as means ± SEM. Significance levels: * and ** indicate significant differences (*P* < 0.05, *P* < 0.01, respectively).

Furthermore, a shRNA sequence targeting VPS28 was employed to suppress its expression in MAC-T cells, which were transfected with the recombinant lentiviral vector LV3. This approach was undertaken to investigate the influence of VPS28 on TG and lipid droplet synthesis. Following transfection, VPS28 expression was found to be significantly reduced by 26.32% in comparison to the control group. This reduction was associated with a marked increase in the protein levels of CD36 and ADFP, which increased by 2.53-fold (*P* < 0.05) and 4.51-fold (*P* < 0.01), respectively (Fig. 2A). Concurrently, a significant enhancement in the TG content of the cells was observed, demonstrating a 1.23-fold increase (*P* = 0.010) (Fig. 2B) in TG was accompanied by a significant rise in the number of lipid droplets (Fig. 2C).

Furthermore, VPS28 knockdown resulted in a 1.48-fold increase in UB levels (*P* < 0.05). This was accompanied by a decrease in the proteasome’s trypsin-like, caspase-like, and chymotrypsin-like components, with a reduction of 19.04% (*P* < 0.05), 12.17% (*P* = 0.070), and 14.19% (*P* = 0.482), respectively (Fig. 2D). These findings highlight the potential of VPS28 knockdown to enhance milk lipid synthesis via the ubiquitination pathway in MAC-T cells.

### VPS28 overexpression promotes ubiquitination pathway in MAC-T cells

To investigate the impact of VPS28 overexpression on milk fat synthesis and the dynamics of the ubiquitination pathway, MAC-T cells were transfected with plasmids designed to increase VPS28 expression. This intervention resulted in a significant elevation in VPS28 level, with a 2.42-fold increase (*P* < 0.01) (Fig. 2A). VPS28 overexpression resulted in a significantly enhancement of CD36 expression by 8.61-fold (*P* < 0.01), while ADFP expression was reduced by approximately 50% compared to the control group (*P* < 0.05). Notwithstanding these alterations in gene expression, the concentration of TG remained unaltered (*P* = 0.050), a finding that was corroborated by Nile Red staining for lipid accumulation (Fig. 2B and C). Moreover, a significant increase in UB levels was observed, increasing by 1.76 times (*P* < 0.01). Additionally, the activities of the proteasome components, including trypsin-like, caspase-like, and chymotrypsin-like. Exhibited notable elevations, reaching 1.84- (*P* < 0.01), 1.63- (*P* < 0.01), and 2.15-fold (*P* < 0.01), respectively (Fig. 2D). These outcomes indicate that VPS28 overexpression influences milk fat synthesis in MAC-T cells, potentially through the modulation of the ubiquitination pathway.

### Proteasome inhibitor EXM increased milk lipid synthesis in MAC-T cells

**Fig. 3:**
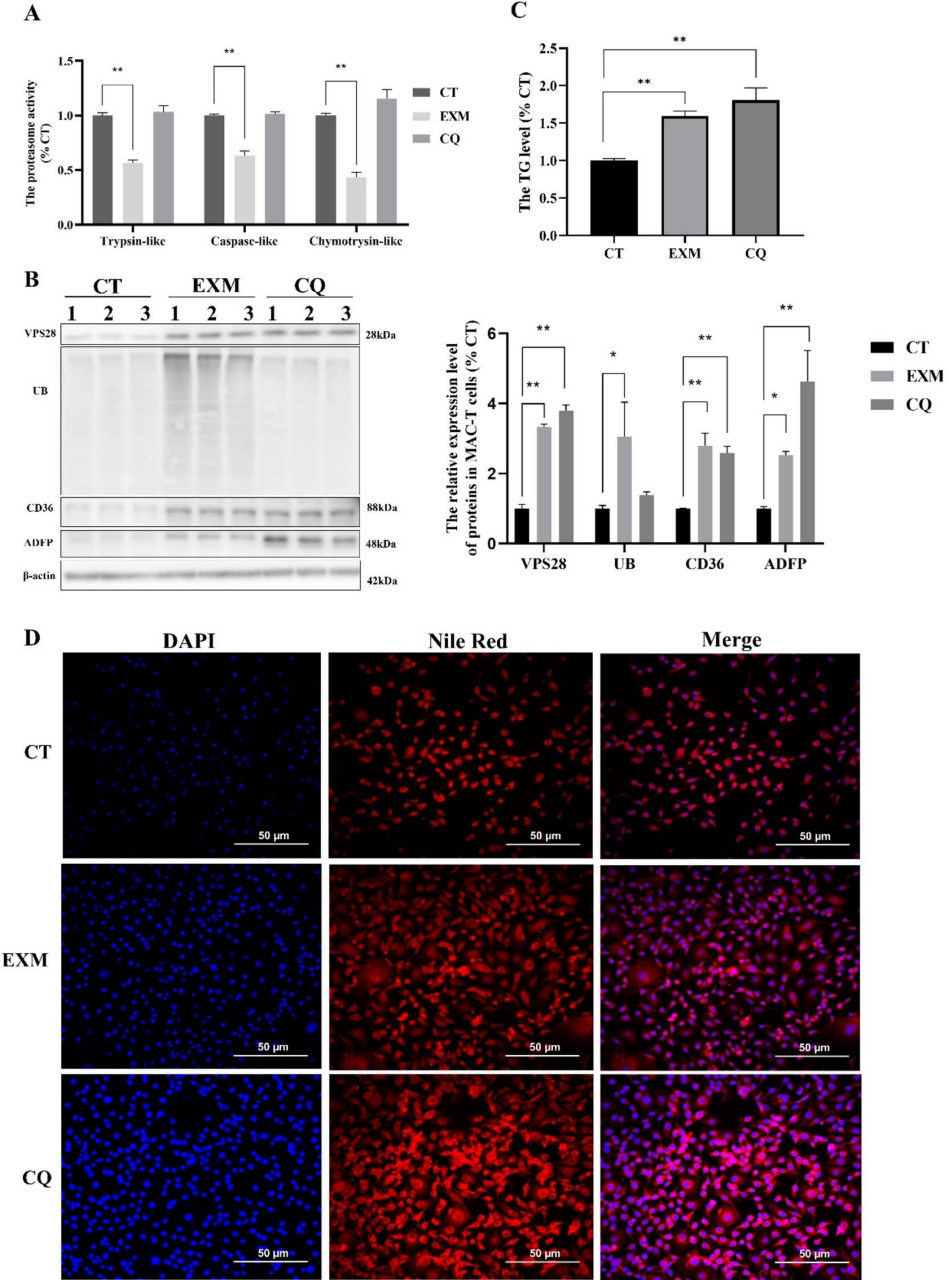
The effect of proteasome and lysosome inhibition in MAC-T cells. (**A**) The three activities of proteasome. (**B**) The relative protein expression levels. (**C**) The relative TG content. (**D**) The Nile Red staining. CT: control. EXM: inhibited proteasome activity using epoxomicin. CQ: inhibited lysosomal activity using chloroquine. All treatments were independently replicated three times. Data are presented as means ± SEM. Significance levels: * and ** indicate significant differences (*P* < 0.05, *P* < 0.01, respectively).

To elucidate the impact of the UPS on milk lipid synthesis within MAC-T cells, we employed the proteasome inhibitor EXM to dampen proteasome activity. Figure 3A shows that proteasome inhibition resulted in reduction in the activities of trypsin-like, caspase-like, and chymotrypsin-like components, with factors of 0.43, 0.30, and 0.57, respectively (all *P* < 0.01). This inhibition was associated with a 3.06-fold increase in UB expression (*P* < 0.05) (Fig. 3B). Furthermore, EXM treatment resulted in a 2.80-fold (*P* < 0.01) increase in CD36 and a 2.52-fold (*P* < 0.05) in ADFP levels (Fig. 3B).

Further analysis, involving the quantification of TG and Nile Red staining (Fig. 3C and D), revealed a 1.36-fold augmentation in TG content (*P* < 0.05), coupled with an accumulation of lipid droplets in the MAC-T cells following EXM treatment. These findings underscore a significant correlation between the UPS and milk fat synthesis in MAC-T cells, highlighting the UPS’s pivotal function in regulating lipid metabolic processes.

### Lysosome inhibitor CQ increased milk lipid synthesis in MAC-T cells

In order to assess the role of ELP in milk lipid synthesis and its contribution to the proteasome-mediated ubiquitination process, the CQ inhibitor was employed to impede ELP activity in MAC-T cells. The results showed that CQ treatment had no significant impact on the proteasome or on UB levels (Fig. 3A, B).

However, as with the observations made with EXM, CQ administration resulted in an upregulation of CD36 and ADFP expressions, with increase of 2.58-fold (*P* < 0.01) and 4.63-fold (*P* < 0.01), respectively. In addition, there was a 1.8-fold elevation in TG content (*P* < 0.01) (Fig. 3C), which was accompanied by an increase in lipid droplet accumulation in MAC-T cells (Fig. 3D). These findings substantiate the pivotal function of ELP in modulating TG metabolism in MAC-T cells, highlighting its involvement in regulating milk lipid synthesis via ubiquitination pathways.

### Intraperitoneal injection of VPS28 knockdown and inhibitors does not have a significant influence on the lifespan of mice

In the in vivo experiment, mice were administered intraperitoneal injections of VPS28 knockdown (using recombinant lentiviral vectors LV3) in conjunction with the proteasome inhibitor EXM and the lysosome inhibitor CQ. The objective of this approach was to ascertain the impact of these interventions on body weight (BW) alterations. Given the observed apoptosis caused by VPS28 overexpression in MAC-T cells, which could potentially result in severe inflammation in mice, we elected not to create a VPS28 overexpression mouse model.

**Fig. 4:**
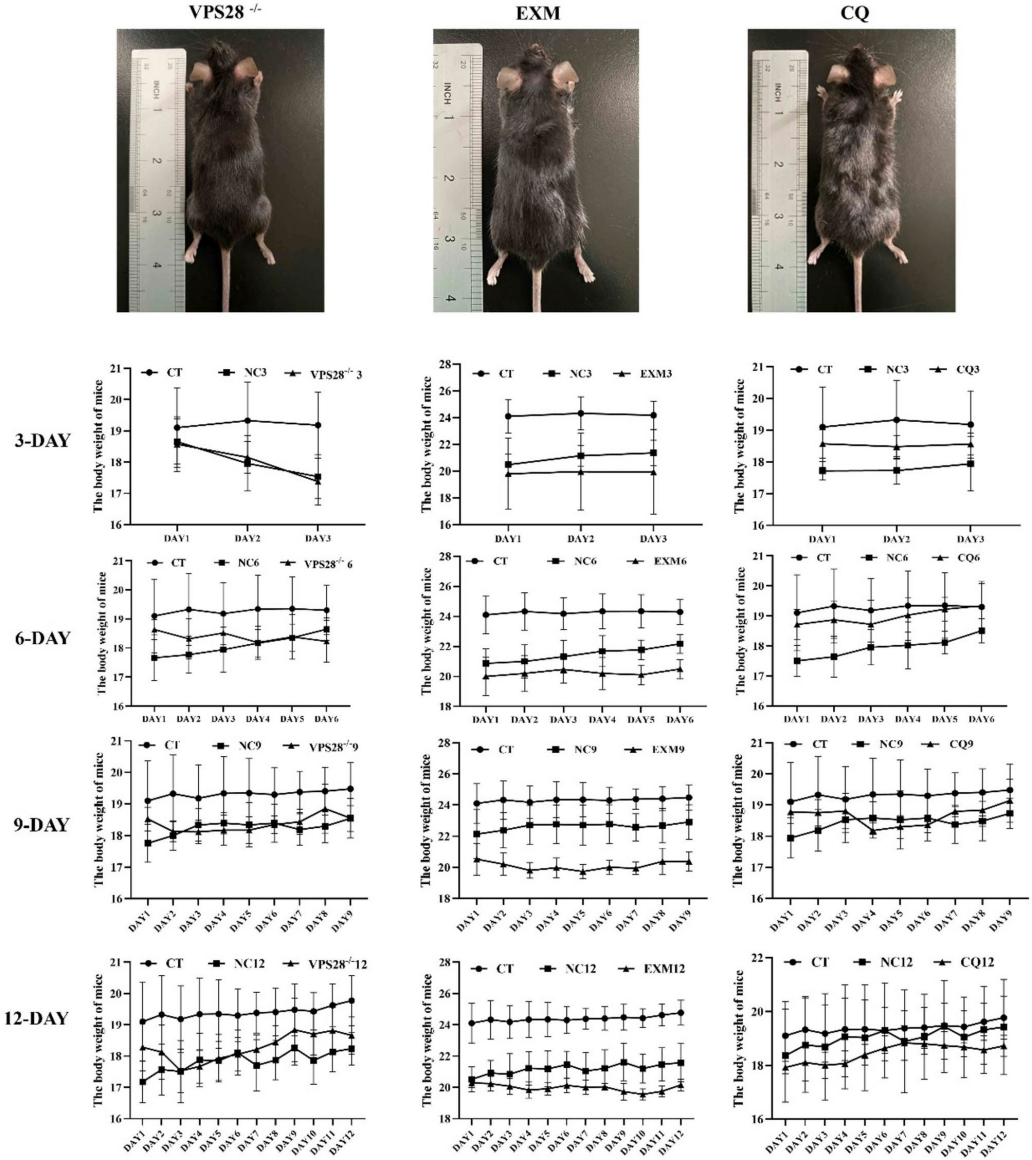
Body weight changes of mice in each group (n = 6 per group). Data was collected by weighing each mouse in the treatment groups at regular intervals throughout the study. The error bars represent SD. Statistical significance was determined using a one-way ANOVA followed by post hoc Tukey’s test.

Our findings revealed that there were no statistically significant differences in BW alterations among the groups (*P* > 0.05) (Fig. 4). This outcome showed that the intraperitoneal injection of the lentivirus or the inhibitors did not result in any notable adverse effects on the mice, indicating the effective establishment of the mouse model for the current study. These results support the viability of using these interventions in vivo without adversely affecting the overall health or BW of the mice, thereby providing a solid foundation for further explorations into the role of VPS28 and ubiquitination pathways in mammalian models.

### Intraperitoneal injection of VPS28 knockdown promote lipid synthesis in both mammary gland and blood in mice

**Fig. 5:**
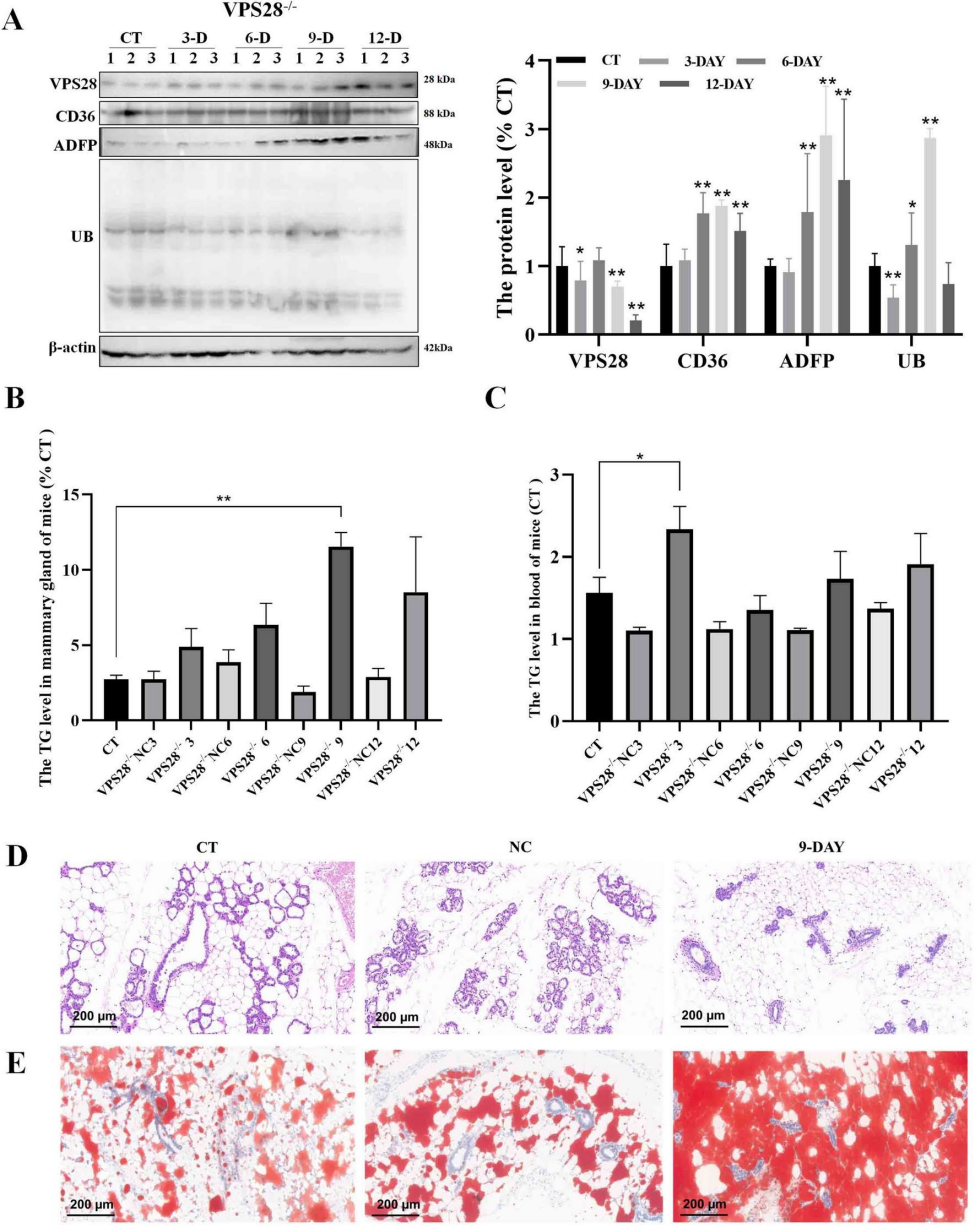
The impact of intraperitoneal injection of VPS28 knockdown on mice. (**A**) Western blot assay was conducted after VPS28 knockdown to assess CD36, ADFP, and UB protein expression in the mammary gland of mice. (**B**, **C**) The levels of TG in the mammary gland and blood of mice were measured (n = 3, error bars represent SEM. Statistical significance was determined using a one-way ANOVA followed by post hoc Tukey’s test). (**D**, **E**) Representative images illustrating the morphology of the mammary gland using HE staining and Oil Red staining were obtained for each treatment group (n = 3 per group).

Following verification of the safety of VPS28 knockdown in mice, an investigation was conducted into its impact on TG synthesis and ubiquitination-related proteins. The initial objective was to assess the levels of VPS28 protein levels in the mammary glands of mice. As shown in Fig. 5A, a notable reduction in VPS28 expression was observed in all treated groups at 3, 9 and 12 days (*P* < 0.05), substantiating the efficacy of the VPS28 knockdown mouse model generated through intraperitoneal lentiviral injection. In comparison to the control group, there were notable increases in CD36, ADFP, and UB expression in mammary gland tissues (*P* < 0.01), indicating that VPS28 knockdown influences TG synthesis and ubiquitination.

The objective was to examine the concentrations of TG in the mammary glands and blood, as well as to conduct lipid droplet staining of the mammary glands. It is noteworthy that VPS28 knockdown results in elevated mammary gland TG levels, with a peak on day 9 (*P* < 0.01), whereas blood TG levels peaked on day 3 (Fig. 5B and C). The 9-day cohort was chosen for a comprehensive lipid droplet analysis in mammary tissues, which revealed an increase in lipid droplets without any observable morphological changes (Fig. 5D and E). These results confirm that VPS28 knockdown influences ubiquitination and regulates TG production in the blood and lipogenesis in mammary glands.

### Intraperitoneal Injection of EXM promote lipid synthesis in both mammary gland and blood in mice

**Fig. 6:**
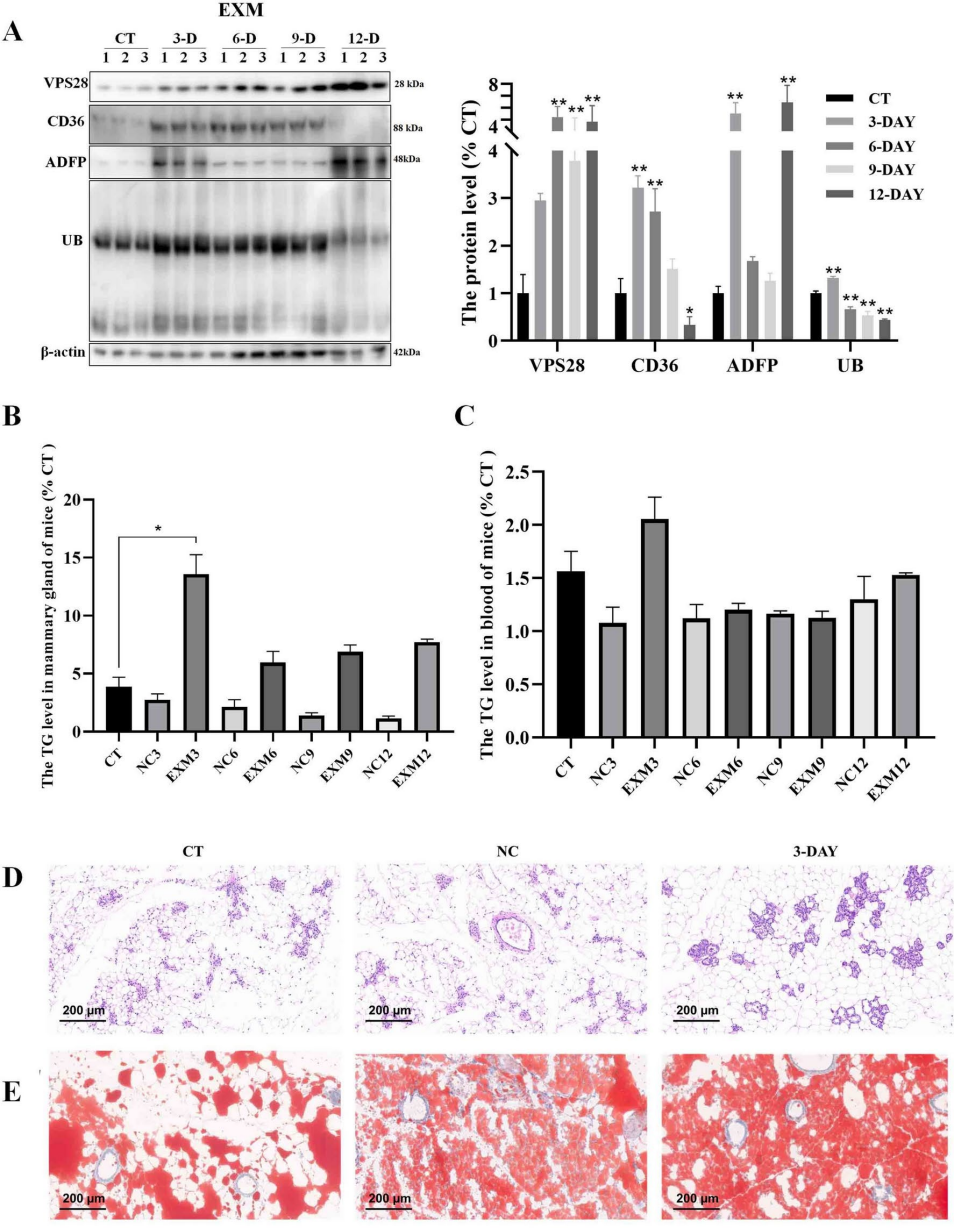
The impact of intraperitoneal injection of EXM on mice was investigated. (**A**) Western blot assay was conducted after EXM injection to assess changes in CD36, ADFP, and UB protein expression in the mammary gland of mice. (**B**, **C**) The levels of TG in the mammary gland and blood of mice were measured (n = 3, error bars represent SEM. Statistical significance was determined using a one-way ANOVA followed by post hoc Tukey’s test). (**D**, **E**) Representative images illustrating the morphology of the mammary gland using HE staining and Oil Red staining were obtained for each treatment group (n = 3 per group).

In mice treated with EXM intraperitoneally, significant alterations in mammary gland protein levels were observed (Fig. 6A). Following EXM injection, there was an incremental increase in VPS28 expression, while CD36, ADFP, and UB levels initially increased before declining. Specifically, there was a significant decrease in CD36 levels on day 12 (*P* < 0.05), while ADFP levels exhibited a substantial increase (*P* < 0.01). TG levels in the mammary glands and blood were measured, showing a peak in mammary gland TG on day 3 post-injection before a decline (*P* < 0.05), with blood TG levels remaining stable (Fig. 6B and C). The 3-day post-EXM treatment model highlighted an increase in mammary gland lipid droplets without tissue morphology alterations (Fig. 6D and E), indicating that EXM-mediated inhibition of the ubiquitin–proteasome pathway significantly boosts milk fat production.

### Intraperitoneal injection of CQ promote lipid synthesis in both mammary gland and blood in mice

**Fig. 7:**
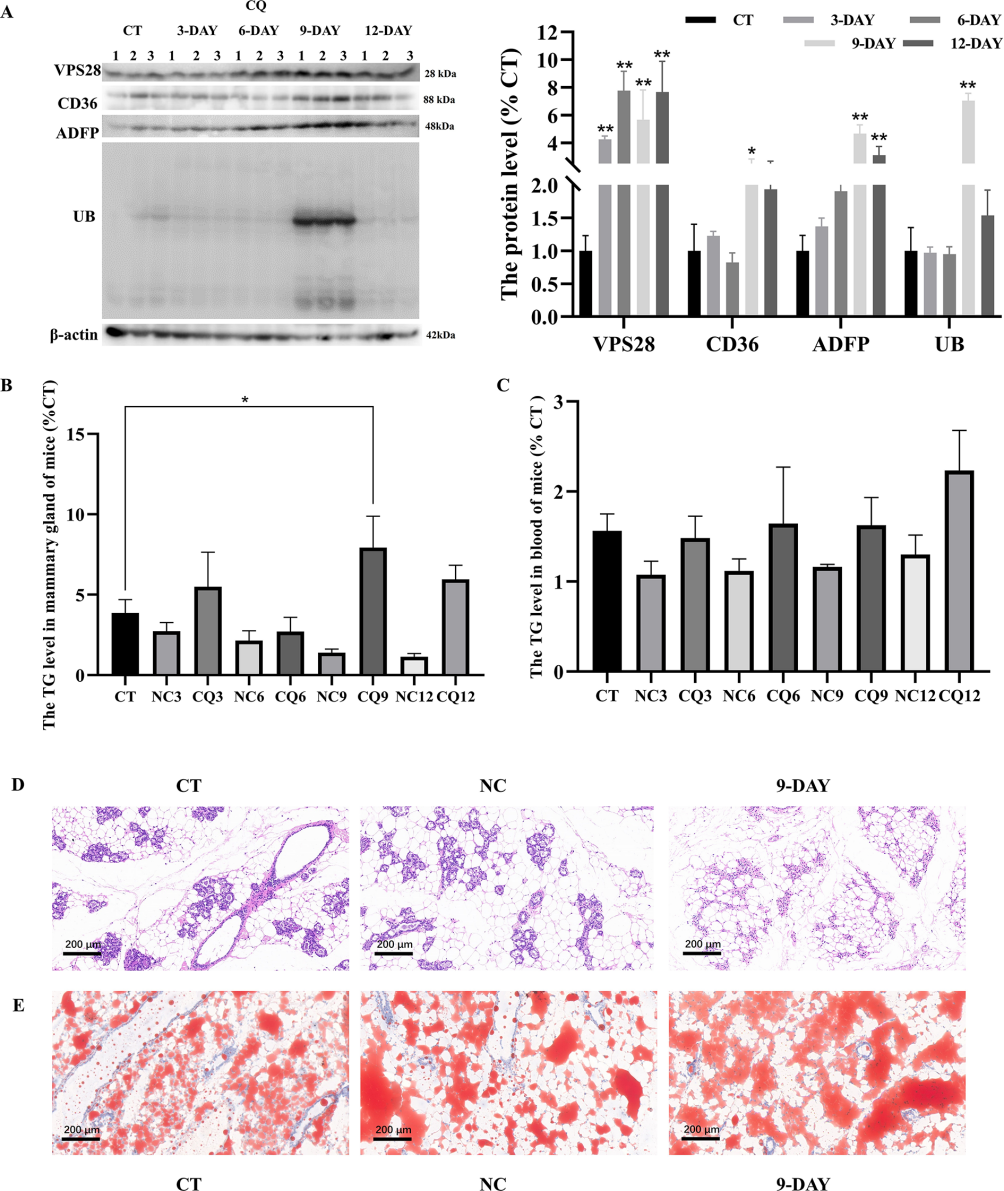
The impact of intraperitoneal injection of CQ on mice was investigated. (**A**) Western blot assay was conducted after CQ injection to assess changes in CD36, ADFP, and UB protein expression in the mammary gland of mice. (**B**, **C**) The levels of TG in the mammary gland and blood of mice were measured (n = 3, error bars represent SEM. Statistical significance was determined using a one-way ANOVA followed by post hoc Tukey’s test). (**D**, **E**) Representative images illustrating the morphology of the mammary gland using HE staining and Oil Red staining were obtained for each treatment group (n = 3 per group).

Similarly, in mice that had been treated with CQ, significant alterations in protein levels were observed in mammary gland tissues following injection (Fig. 7A). Following CQ treatment, significant improvements in VPS28 (*P* < 0.01), CD36 (*P* < 0.05), ADFP (*P* < 0.01), and UB (*P* < 0.01) levels were observed on days 9 and 12 (Fig. 7B). The observed improvements were statistically significant (*P* < 0.01) for VPS28, CD36, ADFP and UB. Furthermore, our findings revealed a peak in mammary gland TG levels on day 9 post-injection, while blood TG exhibited a stable level (Fig. 7C). The 9-day CQ treatment resulted in a notable increase in mammary gland lipid droplets (Fig. 7E), while maintaining normal tissue morphology (Fig. 7D). These findings suggest that the inhibition of the ubiquitin-lysosome pathway using CQ could effectively enhance milk fat production in mouse mammary glands.

## Discussion

VPS28 is a core component of the class E vacuolar protein sorting protein, which is essential for the ESCRT complexes. Our findings revealed that the mutation in VPS28 was associated with reduced expression of the protein and was significantly linked to higher milk fat percentage in Holstein dairy cows^13^. Accordingly, we postulated that the reduced expression of VPS28 may contribute to facilitate milk fat synthesis in bovine. To validate this hypothesis, we identified the regulatory mechanism governing VPS28 expression in MAC-T cell lines and subsequently confirmed these findings in vivo using a mouse model.

To further elucidate the role of VPS28 in milk fat synthesis, we conducted knockdown and overexpression experiments of VPS28 in MAC-T cells and assessed the effects on milk fat synthesis by measuring the expression levels of the lipid synthesis-related genes CD36 and ADFP, TG content, and lipid droplet content. The results showed that reduced expression of VPS28 promoted the expression of the CD36 and ADFP, and also increased the levels of ubiquitinated proteins, TG and lipid droplets in MAC-T cells. VPS28 has been demonstrated to regulate the accumulation of ubiquitinated proteins through the ELP and UPS^23–29^. Therefore, it can be postulated that VPS28 may regulate milk fat synthesis in MAC-T cells by modulating the ubiquitination pathway. The results corroborated our hypothesis that the level of ubiquitinated proteins was elevated following the knockdown and overexpression of VPS28. In addition, we observed that VPS28 knockdown resulted in an accumulation of UB levels and an inhibition of proteasome activity. Furthermore, VPS28 overexpression was found to promote the proteasome activity, indicating that the regulatory impact of VPS28 on milk fat synthesis is partially dependent on ELP- and UPS-mediated ubiquitination pathways.

To investigate the function of ELP and UPS in ubiquitinated membrane proteins and cytoplasmic proteins, the levels of CD36 and ADFP proteins were detected in MAC-T cells. The results demonstrated that the knockdown of VPS28 increased the levels of CD36 and ADFP, while the corresponding overexpression of VPS28 reduced the level of ADFP. Our research has concentrated on TG and lipid droplets in MAC-T cells, which exhibited analogous alterations to those observed for ADFP. These results suggest that the regulation of VPS28 on milk fat synthesis is dependent on the level of ADFP and partially on the level of CD36, which may be mediated by the ubiquitination pathway. The ubiquitinated form of CD36 has been shown to facilitate the transport of FAs into cells, thereby promoting TG synthesis and the formation of lipid droplets^3,36–38^. ADFP is a protein that plays a role in lipid management and is essential for lipid metabolism and storage within cells^11^. Furthermore, it is subject to regulation by the ubiquitination pathway^39,40^. Over the past decade, an increasing body of research has demonstrated the critical role of ELP and UPS in regulating the stability of key players involved in diverse aspects of lipid synthesis, including CD36 and ADFP^32,41^. In the ubiquitination pathway, E3 ubiquitin ligases have been shown to mediate the ubiquitination of substrate proteins, leading to polyubiquitination and subsequent proteosomal degradation. These enzymes have been identified as key regulators of lipid biology. PARKIN is an E3 ligase and target of CD36. The concomitant upregulation of ubiquitinated CD36, PARKIN, and lipid droplets provides evidence that ubiquitination may facilitate the uptake and transport of FAs^22^. Similarly, ADFP is targeted by the E3 ligase MARCHF6 and subsequently involved in the biosynthesis of cellular lipid droplets^41^. These findings suggest that alterations in VPS28 directly influence the levels of ubiquitinated CD36 and ADFP, thereby regulating TG and lipid droplet synthesis in MAC-T cells through the ubiquitination process.

In consideration of the relationship between milk fat synthesis and ELP or USP, we introduced the lysosomal inhibitor CQ and the proteasomal inhibitor EXM to MAC-T cells. CQ, a medication commonly used to treat malaria and autoimmune conditions, impedes the lysosomal fusion process and modifies the acidic environment within lysosomes^42^. This disruption affects the distribution and breakdown of substances within cells, as evidenced by previous studies^43^. Moreover, research suggests that CQ may impact autophagy, a cellular degradation process, which could influence the clearance and processing of cellular debris^44^. EXM targets key proteins within the proteasome, specifically binding to the active sites of the 20S proteasome, thereby obstructing the proteasomal degradation route and hindering the breakdown of ubiquitinated proteins within cells^45,46^. It has been demonstrated that the inhibition of ubiquitination signaling pathways can result in the accumulation of ubiquitinated CD36 and ADFP^3,47^. This accumulation may be the result of post-translational regulation by the ubiquitination pathway, particularly in the context of proteasome inhibition. In MAC-T cells, our results demonstrated that both CQ and EXM did not affect the cell viability, yet they enhanced the expression of VPS28, CD36, and ADFP. This was accompanied by a significant rise in TG content and the accumulation of lipid droplets. It is postulated that these effects may be attributable to the ubiquitination pathways mediated by ELP and USP, thereby highlighting the interconnection between milk fat synthesis and the ELP or UPS.

The study of milk fat synthesis in cows inevitably gives rise to significant financial and managerial challenges, which must be considered in the planning and execution of any research project in this field. For this reason, Chandler developed and characterized an experimental mouse model of the mammary gland as early as the 1970s^48–50^. Mice remain an effective model for studying bovine mammary gland physiology due to similarities in mammary gland structure: both species possess functionally and anatomically independent mammary glands, with mice also having inguinal mammary glands. Additionally, histological changes observed in mouse mammary tissue can serve as a useful proxy for changes in cows, making it a valuable model for exploring milk fat synthesis mechanisms.

Therefore, we inhibited VPS28 expression via intraperitoneal injection of lentivirus, thereby demonstrating that knockdown of VPS28 in mice promoted milk fat synthesis and the ubiquitination levels. These outcomes provide compelling evidence to support the hypothesis that VPS28 directly influences TG and lipid droplet synthesis through the ubiquitination process. These findings in accordance with our previous discovery. Furthermore, we employed CQ and EXM to inhibit lysosomal and proteasomal activity, respectively. The effects of CQ and EXM treatment revealed no adverse effects on the mammary gland tissues of mice revealed no adverse effects, indicating that the treatment was safe at the utilized concentrations. Concomitantly, there was a notable elevation in the levels of VPS28, CD36, and ADFP. It is noteworthy that, in comparison to the knockdown of VPS28, a more pronounced positive impact on TG levels and lipid droplet accumulation were observed when CQ or EXM was administered. This finding not only corroborates VPS28’s regulatory capacity on TG and lipid droplet synthesis via the UPS but also highlights the UPS’s central role in TG and lipid droplet production. Furthermore, the data indicated that reduced VPS28 expression and the addition of CQ or EXM to dairy cow feed may improve health and mammary function. However, further exploration is necessary to confirm this hypothesis. Additionally, the combined results of cell activity in MAC-T cells and mammary tissue morphology in mice indicated that reduced VPS28 expression and the addition of CQ or EXM to dairy cow diets may improve health and mammary function.

## Conclusions

The present study demonstrated that in MAC-T cells, VPS28 knockdown promotes milk fat synthesis by regulating the ubiquitination pathways and targeting ubiquitinated CD36 and ADFP. In this study, we successfully knocked down VPS28 in MAC-T cells and mice and observed a significant increase in CD36 and ADFP levels, as well as a facilitation of TG synthesis and ubiquitination levels. Similar results were observed when the ubiquitin-proteasome system and the endosomal-lysosomal pathway inhibitors EXM and CQ were employed. The function of VPS28 in milk fat synthesis was elucidated and the potential utility of EXM in promoting milk fat synthesis was verified. The results of this study could contribute to a greater understanding of the regulatory network of milk fat synthesis in a bovine in vitro system.

## Materials and methods

In order to verify the regulation of VPS28 on TG through the ubiquitination pathway in vitro and in vivo. To investigate the role of VPS28 in TG regulation, a knockdown and overexpression of the VPS28 gene was conducted in MAC-T cells. Additionally, an incubation with EXM and CQ was performed. Subsequently, an intraperitoneal injection with VPS28-knockdown lentivirus, EXM and CQ were performed in female C57BL/6 mice. Subsequently, western blot, cell viability analysis, Nile Red staining, TG content analysis, HE staining, and Oil Red O staining were conducted.

### Reagents

CQ (CAS No. C6628-25G) was purchased from Sigma (Sigma-Aldrich, USA), EXM (CAS No. 134381-21-8) and Nile Red (CAS No. 7385-67-3) were purchased from Med Chem Express (MCE, USA). Antibodies were purchased from Abcam (Cambridge, UK). All other reagents were purchased from Life Technologies (Carlsbad, CA, USA) unless noted otherwise. EXM and CQ were dissolved in a solution comprising 10% DMSO and 90% saline with 20% SBE-β-CD to use.

### Cell culture and treatments

The MAC-T cells were donated by the Chinese Academy of Sciences, Kunming Institute of Zoology. The cells first cultured in serum-containing medium DMEM (CAS No. 11965092), supplemented with 10 kU/mL penicillin, 10 mg/mL streptomycin, and 10% FBS (fetal bovine serum, CAS No. A5256701). All cells were cultured in plastic cell culture plates at 37℃ in a humidified atmosphere containing 75% CO_2_.

VPS28 was knocked down using recombinant lentiviral vector LV3 (H1/GFP&Puro) containing RNA interference fragments (shRNA: CCGGGGACGTGGTCTCGCTCTTTATCTCGAGATAAAGAGCGAGACCACGTCCTTTTTG), and overexpressed using recombinant lentiviral vectors pCDH-CMV-MCS-EF1-GFP-Puro-VPS28. The detailed procedures as following:

1. Prepare a lentivirus in 293T cells, including 293T cells, recombinant lentiviral vectors, packaging plasmids (psPAX2 and pMD2.G), and a transfection reagent, Lipofectamine 3000.
2. One day prior to the transfection procedure, it is necessary to seed the 293T cells in a culture plate until they reach a confluence of 70–80% at the time of transfection.
3. The plasmid mixture should be prepared by combining the lentiviral vector, psPAX2, and pMD2.G in an optimal ratio of 4:3:1. The plasmids must then be mixed with the transfection reagent and incubated for 15 min to allow complex formation. Subsequently, the complex should be added to the 293 T cells with gentle swirling to ensure thorough mixing. The cells should be incubated at 37 °C in a 5% CO₂ environment for a period of 6–8 h.
4. Thereafter, the medium should be replaced with fresh culture medium in order to enhance the yield of the virus.
5. To obtain the virus, the cell culture supernatant should be harvested at 48 and 72 h post-transfection. It is then necessary to filter it through a 0.45 µm filter in order to remove any cell debris. Finally, the virus can be concentrated using ultracentrifugation in order to obtain a high-titer virus (2 × 10^8^ plaque-forming units per mL).
6. Following a 72-h incubation period with lentivirus, the MAC-T cells in the six-well plates were examined to ascertain the efficacy of infection and identify any morphological alterations.
7. Subsequently, the cells were meticulously rinsed with phosphate-buffered saline (PBS) to eliminate any residual viral particles. Subsequently, the wells were replenished with fresh complete growth medium, and the cells were cultured for an additional 48 h to allow for optimal expression of the target gene. To evaluate the efficacy of the infection process, fluorescence microscopy was employed to identify the presence of GFP introduced by the lentiviral constructs.
8. Subsequently, the cells were prepared for protein extraction.
9. Proteasome-inhibited MAC-T cells were cultured with 1 μmol/L EXM. Lysosome-inhibited MAC-T cells were cultured with 50 μmol/L CQ. Control cells were treated with solution of EXM and CQ. Following a 24-h incubation, all cells were collected for protein extraction, as well as analysis of TG.

### Mice

The in vivo experiments were performed with 150 female C57BL/6 mice (purchased from Yunnan University of Traditional Chinese Medicine), aged 7–8 weeks old, accommodated at the Kunming Institute of Zoology, Chinese Academy of Sciences, located in Kunming, China. The animals were kept under controlled environmental conditions at a temperature of 25 °C with a humidity level set at 55%. The animals were provided with 12 h of ambient lighting in ventilated and pathogen-free cages. The animals were provided with unrestricted access to drinking water and food and their health status was monitored on a daily basis. The study was conducted in accordance with the ethical standards set forth by the Academic Committee of Southwest Forestry University (Approval ID: SWFU-20220307-1) for the use and care of animals. We confirm that all experimental procedures and protocols conducted in this study were performed in accordance with the relevant guidelines and regulations, including the ARRIVE guidelines for reporting animal research. The study protocol was approved by the Academic Committee of Southwest Forestry University.

### Treatments

The mice were randomly assigned to one of four groups: a control group (n = 6), a VPS28 knockdown group (n = 48, 100 μL per day, with a lentiviral titer of 2 × 10^8^ plaque-forming units per mL), an EXM model group (n = 48, 5 μM per day, with a concentration of 1 mg/mL), and a CQ model group (n = 48, 3.1 mM per day, with a concentration of 1.6 mg/mL). The three model groups were established through the continuous intraperitoneal administration of VPS28-knockdown lentivirus over a 12-day period. Subsequently, each model group was randomly divided into 8 smaller groups, with 6 mice in each group. The experimental groups were designated as follows: 3-day group (n = 6 each), 6-day group (n = 6 each), 9-day group (n = 6 each) and 12-day group (n = 6 each). Each of these groups was further subdivided into a negative control (NC) group, which consisted of six mice. The NC groups for the EXM and CQ groups were treated with a solution containing 10% dimethyl sulfoxide (DMSO) and 90% saline, supplemented with 20% sulfobutylether-β-cyclodextrin (SBE-β-CD). The NC group for the VPS28 knockdown group was treated with an empty viral vector. Throughout the study, all mice were provided with a standard chow diet (SWS9102-1010086, Jiangsu Synergetic Pharmaceutical Bioengineering Co., LTD, China) and had access to drinking water.

The body weight (BW) of the mice was meticulously recorded on a daily basis throughout the entire study period in order to monitor any fluctuations resulting from the administration of the test substance. This methodology was established in a previous study and was employed as a means of assessing the growth performance parameters of the mice.

Following injection, blood samples were collected from the eyes of six mice per group for sample collection. Subsequently, the mice were euthanized by cervical dislocation, and samples of the mammary gland were obtained post-sacrifice. For the purpose of histomorphology analysis, the mammary glands of three mice from each group were fixed in a 4% polyformaldehyde solution for a period of 24 h.

The remaining tissues from the other three mice per group were washed with ice-cold PBS for the analysis of TG content. Furthermore, all other tissues were collected and stored at − 80 °C for subsequent western blot analysis. The methods are consistent with the method used for the cell samples.

### Western blot analysis

Following their treatment in accordance with the experimental assignments, the MAC-T cells or mammary glands from mice were lysed in cold lysis buffer at 4 °C for 30 min, broken by ultrasonic waves, and then subjected to centrifugation at 12, 000 × g for 20 min. The supernatants were collected, and the protein was quantified using a bicinchoninic acid (BCA) protein assay kit (Beyotime Biotechnology, China). Western blotting was employed to detect the levels of VPS28 (No. ab154793, 1:500), CD36 (No. ab252922, 1:1,000), ADFP (No. ab181452, 1:1,000), and UB (No. sc-53509, Santa Cruz, USA, 1:500). Anti-β-actin (No.66009, Proteintech Group, USA, 1:1,000) used as an internal control, the HRS-conjugated secondary antibody was purchased from Cell Signaling Technology (Danvers, MA, USA, No. 7076S). The bands were detected using a chemiluminescent ECL system (Promega, USA) and quantified with Image J (version 1.8.0)^51^.

### TG content analysis

To analyze TG content, cellular and tissue samples underwent extraction and quantification using a TG assay kit (CAS: A110-1-1, Nanjing Jiancheng Bioengineering Institute, China) according to the method described in a previous study^52^. Briefly, after the addition of PBS, the cells were subjected to ultrasonic disruption for 300 s, followed by the addition of a working solution and a 30-min reaction period. Subsequently, the absorbance was measured using an enzyme-linked immunosorbent assay (ELISA) reader at a wavelength of 510 nm. Concurrently, the total protein concentration in the cells or tissues was determined using BCA, and the TG content in the cells and tissues was calculated using the following formula:

$${\text{TG}}\;{\text{content }}\left( {\frac{{{\text{mmol}}}}{{{\text{gprot}}}}} \right) = \frac{{OD_{sample} { } - { }OD_{control} }}{{OD_{standard} - OD_{control} }} \times \frac{{{\text{Standard}}\;{\text{concentration}}\;\left( {{\text{mmol}}/{\text{L}}} \right)}}{{{\text{Sample}}\;{\text{protein}}\;{\text{concentration}}\;\left( {{\text{gprot}}/{\text{L}}} \right)}}$$

### Nile Red staining

The Nile Red staining was also performed on MAC-T cells according to the instructions provided by the manufacturer. The cells were seeded into 12-well plates and allowed to culture for 24 h. After that, the cells were fixed with 4% paraformaldehyde for 15 min at room temperature and washed three times with 1% BSA in PBS. Subsequently, the cells were incubated with 10 µg/mL Nile Red at 4 °C for 15 min and washed three times with 1% BSA in PBS. The stained cells were then incubated with DAPI (No. P0131, Beyotime Biotechnology, China) until air-dried naturally. Finally, the cells were covered with a cover slip and the lipids were visualized using a confocal microscope (Olympus IX81-FV1000) to capture images.

For Nile Red staining of tissue, fresh mammary gland tissue was fixed in a 4% paraformaldehyde solution for 24 h. Subsequently, the tissue was embedded in OCT (Optimal Cutting Temperature) and 8-μm-thick sections were obtained for staining. The methodology employed used for the tissue samples is analogous to that utilized for the cell samples.

### Oil Red O staining

Frozen sections, with a thickness of 8 μm thick, were utilized for Oil Red O staining. The staining process entailed the preparation of an Oil Red O working solution with a concentration of 0.5–5% (w/v), which was achieved by dissolving the powder in isopropanol. Subsequently, the tissue sections were incubated in the solution for 10–20 min at room temperature, followed by a brief rinse in distilled water to remove excess stain. The microscopic observation of mammary gland alveoli and fat deposition was conducted in accordance with the established protocol.

### Hematoxylin–eosin (HE) staining

The fresh mammary gland tissue was fixed in a 4% paraformaldehyde solution for a period 24 h. Subsequently, the tissue was embedded in paraffin, and 8-μm-thick sections were obtained and subjected to staining in accordance with the following steps: (1) Dewaxing: The sections were treated with xylene for 5 min and repeated thrice, followed by rinsing with anhydrous ethanol for 5 min and repeating the rinse thrice. (2) Ethanol Gradient Elution: The sections were sequentially washed with 95%, 80%, 75% ethanol, and distilled water for 2 min each. (3) Hematoxylin Staining: The sections were stained with hematoxylin drops for 5 min, followed by rinsing with tap water for 2 min. (4) Differentiation: The sections were differentiated with 1% hydrochloric acid ethanol for 30 s, followed by soaking in antiblue treatment for 15 min. (5) Eosin Staining: The sections were stained with Kay red dye drops for 2 min, followed by rinsing with tap water for 3–5 s. (6) Dehydration: Dehydration of the sections was carried out using 70% ethanol, 80% ethanol, 95% ethanol, 100% ethanol (twice), and xylene (thrice), each for 1 min. (7) Sealing: Finally, neutral resin was applied for sealing. Microscopic examination and image acquisition were performed using an orthotopic light microscope (Nikon Eclipse E100, Nikon, Japan) equipped with an imaging system (Nikon DS-U3, Nikon, Japan)^53^. Photomicrographs of three sections per mouse were captured at a magnification of 200×.

### Proteasome activity analysis

The proteasome activities were analyzed by measuring cells using the Proteasome-Glo™ Chymotrypsin-Like, Caspase-Like, and Trypsin-Like Cell-Based Assays (No. G1180, Promega, USA), following the methodology proposed in a previous study^51^. Briefly, the cells were lysed in a buffer containing a proteasome inhibitor, and the resulting lysates were added to the substrate mixtures supplied in the kit. The mixtures contained luminogenic peptide substrates that were specific for the chymotrypsin-like, caspase-like, or trypsin-like activity of the proteasome. Following a 15-min incubation period at room temperature, the luminescence was measured using a microplate reader.

### Cell viability analysis

The CCK-8 method was utilized for purpose of conducting a cell viability. MAC-T cells were seeded in a 96-well cell culture plate and allowed to reach 70% confluence, as determined by microscopy. Subsequently, the cells were treated with VPS28 knockdown, VPS28 overexpression, EXM solution, and CQ solution, as previously described. Subsequently, following the designated transfection and incubation period, the culture medium was replaced with 10% CCK-8 basic medium, and the cells were further incubated for 3 h. The absorbance of each cell group was determined at a wavelength of 450 nm using an automated microplate reader. The cell viability was determined based on the absorbance values obtained from the assay^54^.

### Statistical analysis

Each treatment was performed in triplicate, and the resulting data were expressed as means ± standard error of the means (SEM). The densitometry values of western blots were quantified using Image J software (version 1.8.0), and protein abundance was normalized to β-actin. The data were subjected to analysis using analysis of variance and multiple testing with SPSS Statistics software (version 22.0), and graphs were generated with GraphPad Prism 8.0.1 software (GraphPad, San Diego, CA, USA). Significant differences were considered to be those with a *P* value of less than 0.05, and highly significant differences were considered to be those with a *P* value of less than 0.01.

## Supplementary Information

Supplementary Information.

## Data Availability

All data measured or analyzed during this work are available from the corresponding author upon reasonable request.

## Abbreviations

VPS28: Vacuolar protein sorting 28
MAC-T: Bovine mammary epithelial cell line
CD36: CD36 molecule
ADFP: Adipose differentiation-related protein
TG: Triglyceride
UB: Ubiquitin
UPS: Ubiquitin–proteasome system
ELP: Endosomal-lysosomal pathway
ESCRT: Endosomal sorting complexes required for transport
EXM: Epoxomicin
CQ: Chloroquine
HE: Hematoxylin–eosin
BW: Body weight
FBS: Fetal bovine serum
NC: Negative control
WB: Western blot
OCT: Optimal cutting temperature
