# Author Correction: VPS28 regulates triglyceride synthesis via ubiquitination in bovine mammary epithelial cells

**DOI:** 10.1038/s41598-025-98482-2

**Published:** 2025-05-01

**Authors:** Lily Liu, Jinhai Wang, Xianrui Zheng, Qin Zhang

**Affiliations:** 1 College of Biological and Food Engineering, Southwest Forestry University, Kunming, 650224 China; 2 The Roslin Institute, University of Edinburgh, Edinburgh, EH25 9RG UK; 3 College of Animal Science and Technology, Anhui Agricultural University, Hefei, 230036 China; 4 College of Animal Science and Technology, Shandong Agricultural University, Tai’an, 271018 China

Correction to: *Scientific Reports* 10.1038/s41598-024-82774-0, published online 28 December 2024

The original version of this Article contained an error in Figure 7 where the images were mistakenly duplicated from Figure 5 and Figure 6. The original Figure 7 and accompanying legend appear below.

**Fig. 7:**
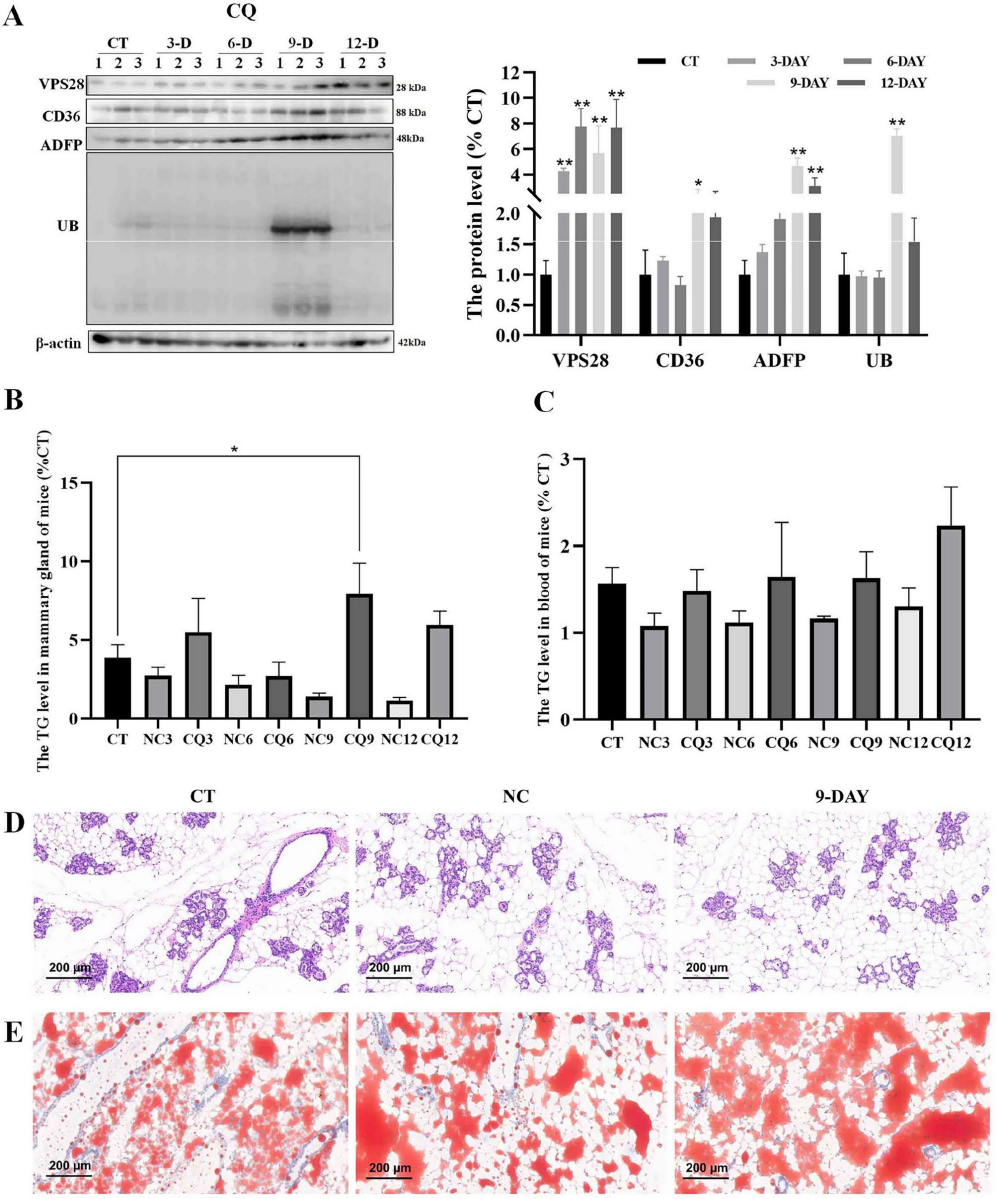
The impact of intraperitoneal injection of CQ on mice was investigated. (**A**) Western blot assay was conducted after CQ injection to assess changes in CD36, ADFP, and UB protein expression in the mammary gland of mice. (**B**, **C**) The levels of TG in the mammary gland and blood of mice were measured (n = 3, error bars represent SEM. Statistical significance was determined using a one-way ANOVA followed by post hoc Tukey’s test). (**D**, **E**) Representative images illustrating the morphology of the mammary gland using HE staining and Oil Red staining were obtained for each treatment group (n = 3 per group).

The original Article has been corrected.

