# Supplementary material for: VPS28 regulates triglyceride synthesis via ubiquitination in bovine mammary epithelial cells
Source: Sci Rep. 2024 Dec 28;14:31310. doi: 10.1038/s41598-024-82774-0 (PMC11682384; doi:10.1038/s41598-024-82774-0)
Supplement: Supplementary file 1 — Supplementary Information. [file 41598_2024_82774_MOESM1_ESM.docx]

**The blots of VPS28 in MAC-T cells.**


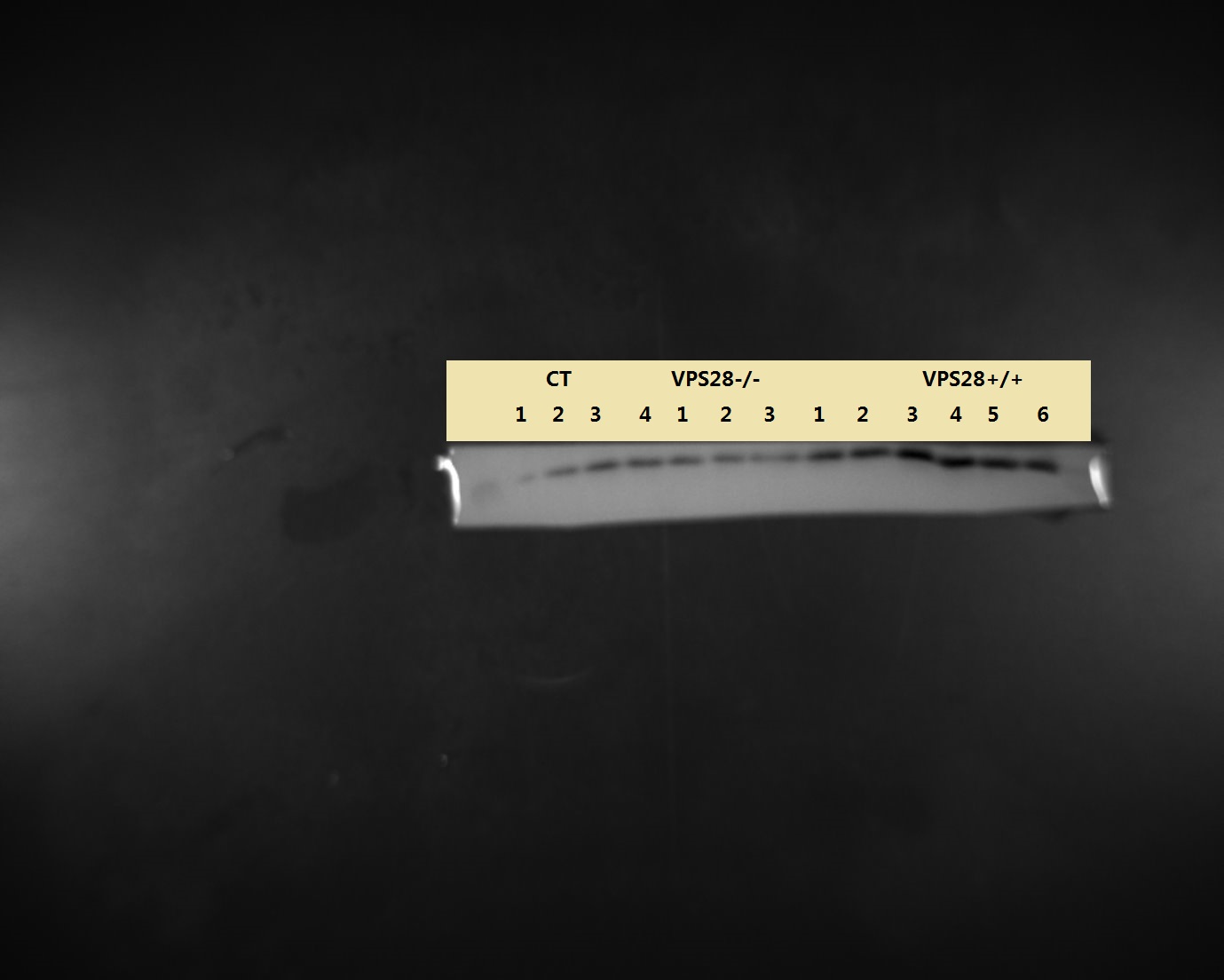


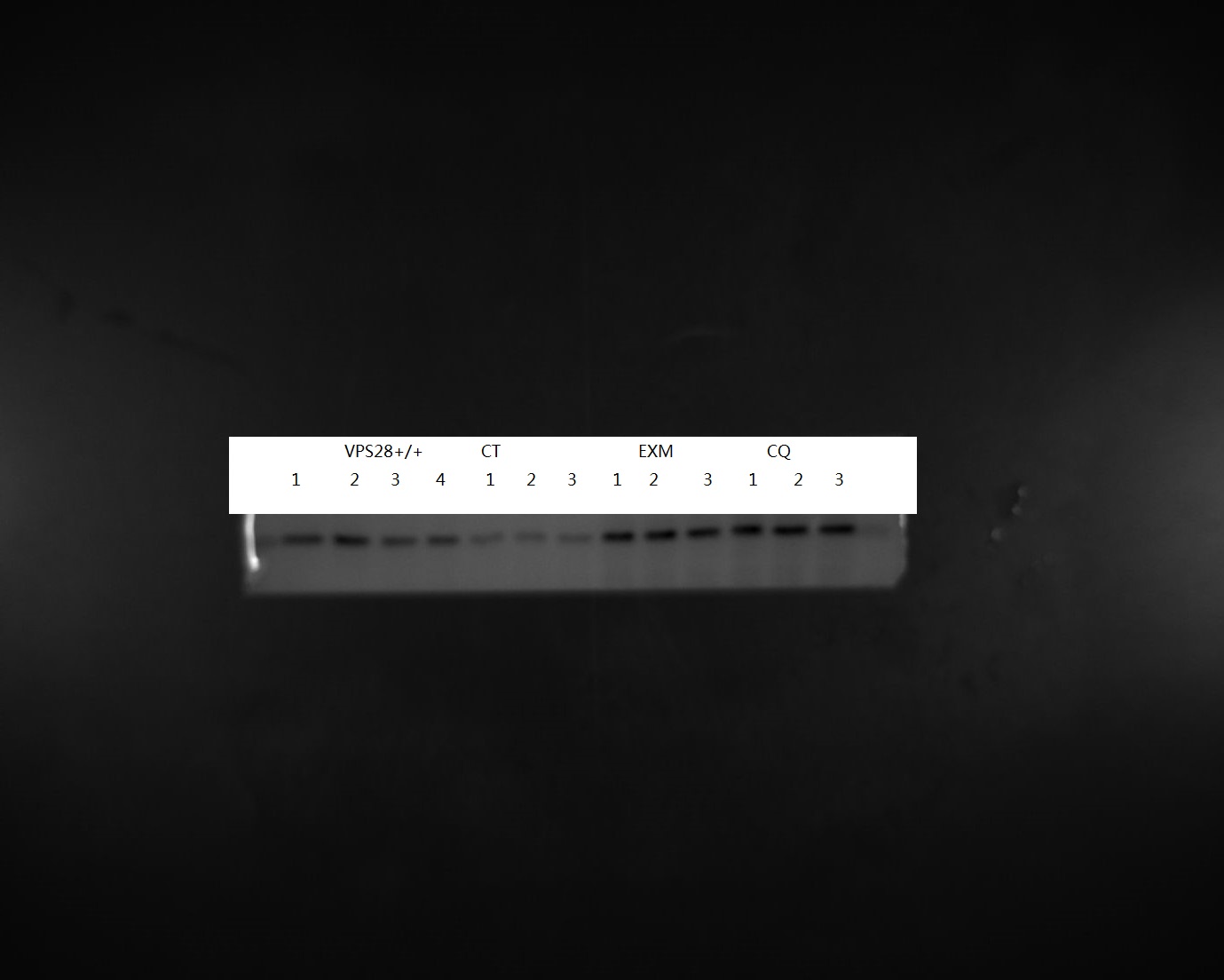


**The blots of VPS28 in mice.**

**VPS28-/-**


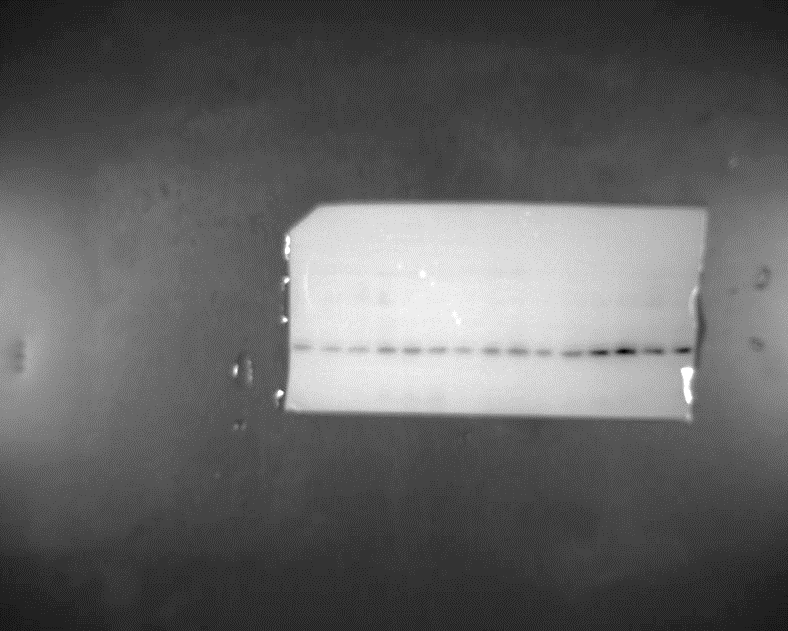


**CT 3-DAY 6-DAY 9-DAY 12-DAY**

**1 2 3 1 2 3 1 2 3 1 2 3 1 2 3**

EXM


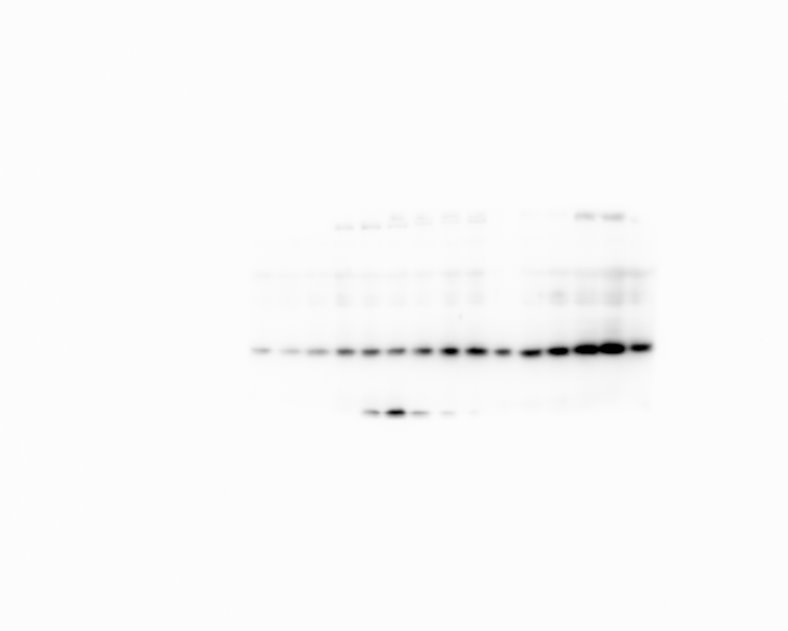


**CT 3-DAY 6-DAY 9-DAY 12-DAY**

**1 2 3 1 2 3 1 2 3 1 2 3 1 2 3**

CQ


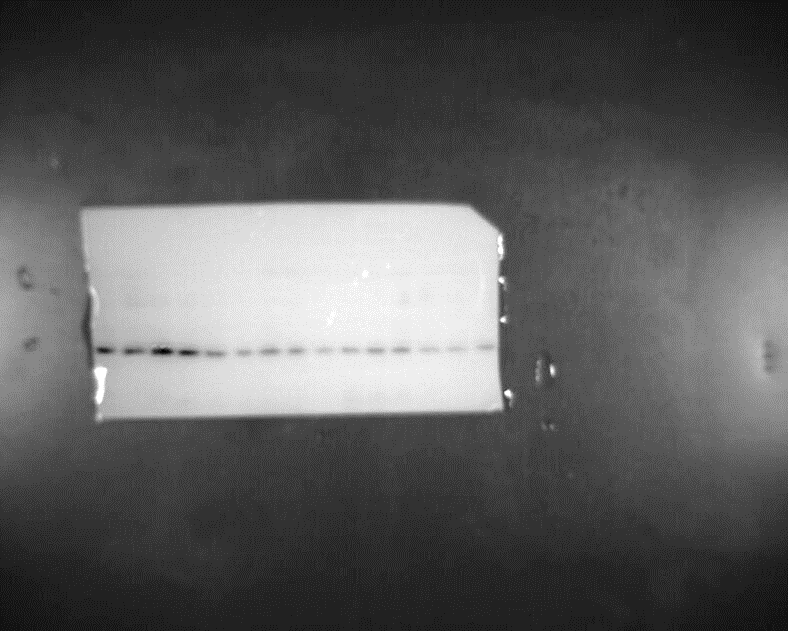


**CT 3-DAY 6-DAY 9-DAY 12-DAY**

**1 2 3 1 2 3 1 2 3 1 2 3 1 2 3**

**The blots of UB in MAC-T cells.**


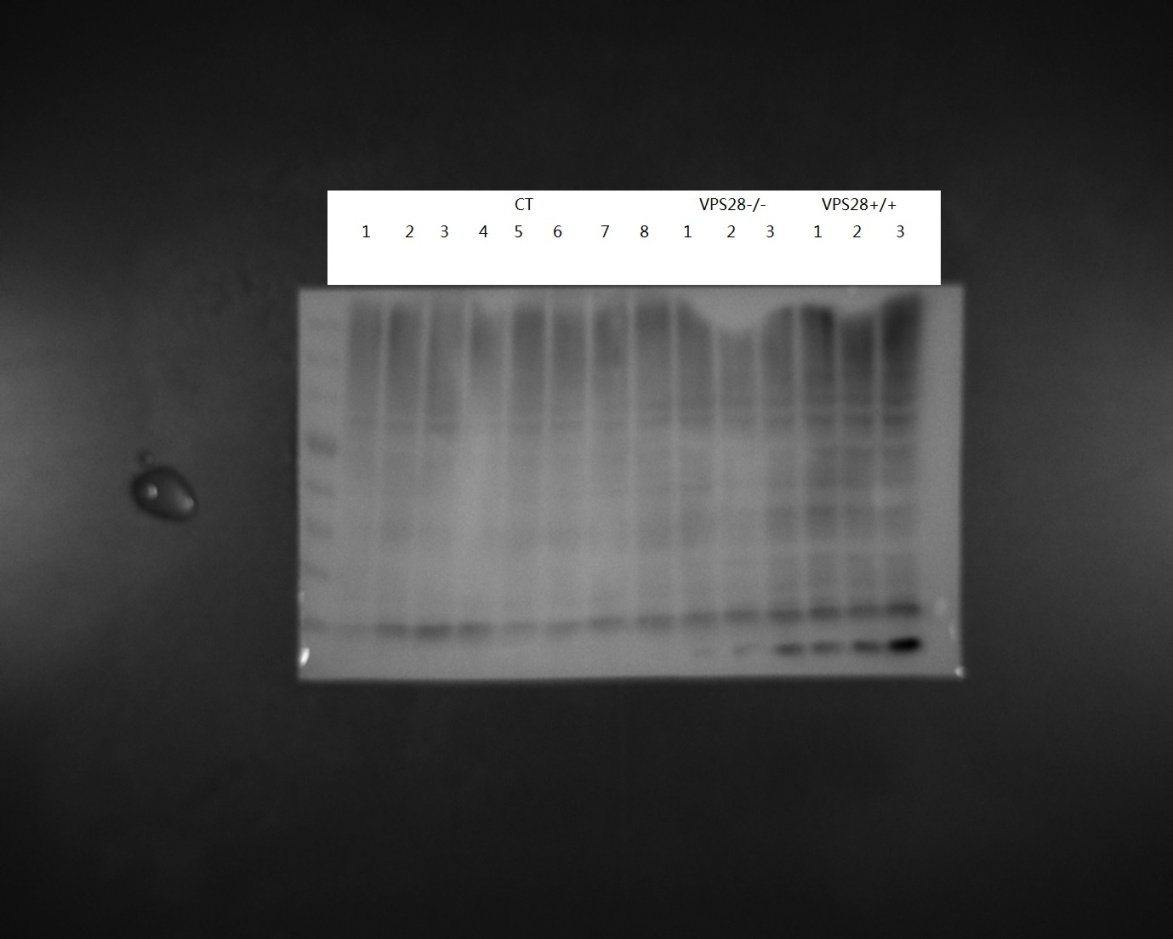


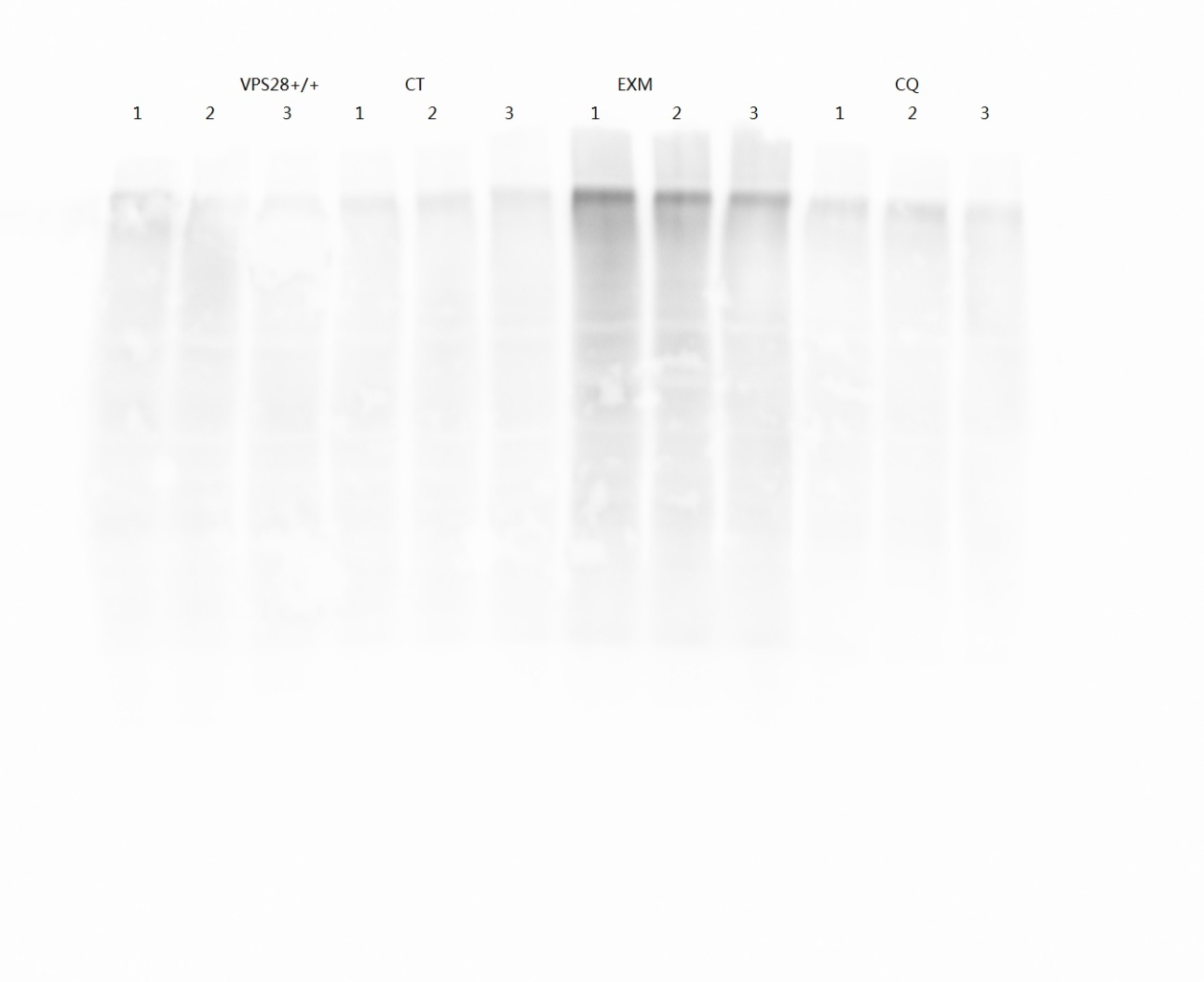


**The blots of UB in mice.**

**VPS28-/-**


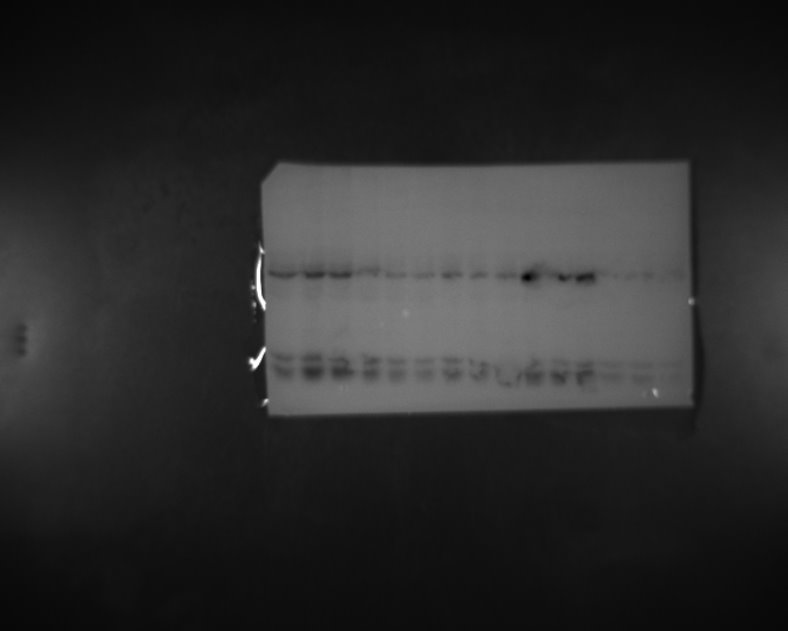


**CT 3-DAY 6-DAY 9-DAY 12-DAY**

**1 2 3 1 2 3 1 2 3 1 2 3 1 2 3**

**EXM**


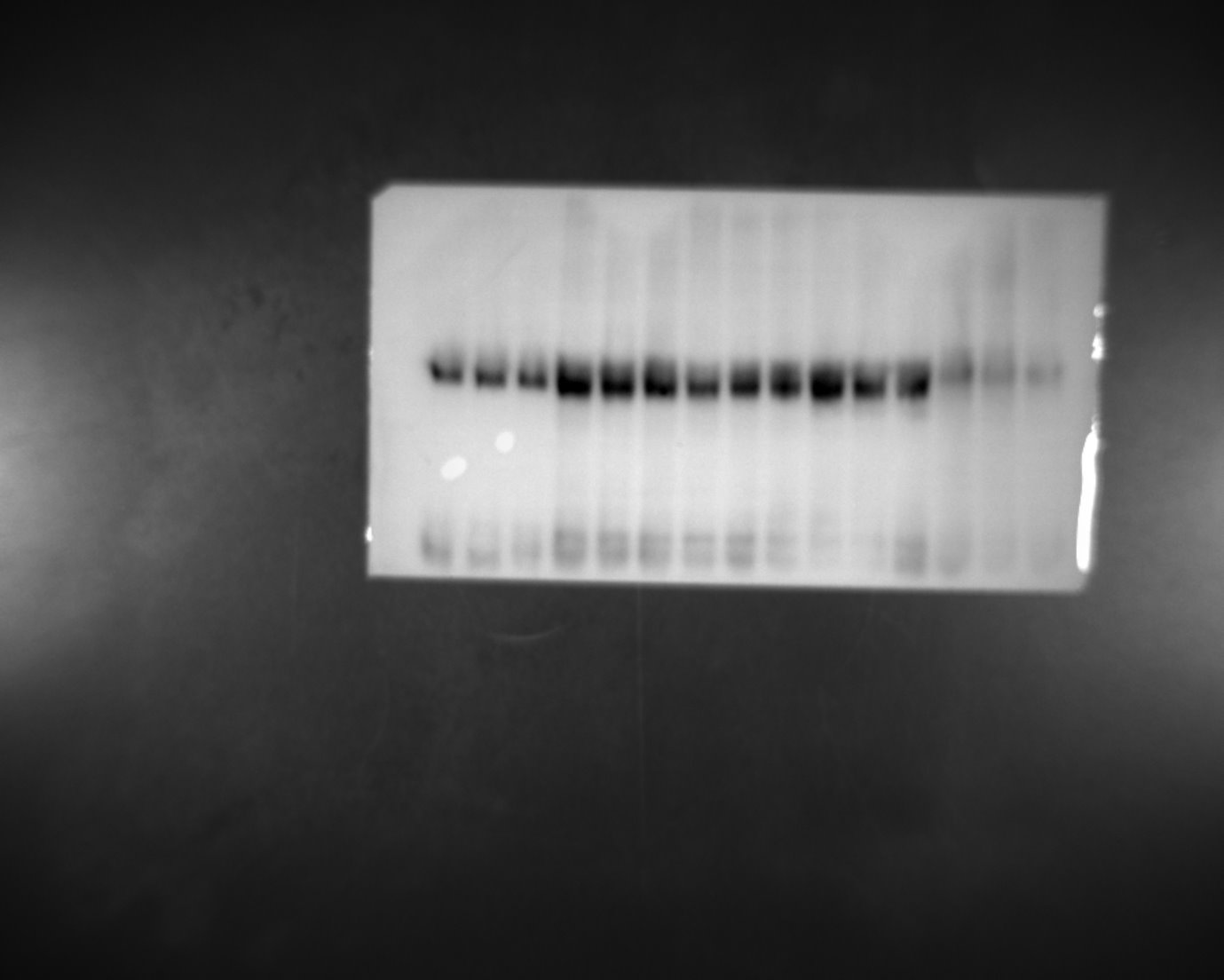


**CT 3-DAY 6-DAY 9-DAY 12-DAY**

**1 2 3 1 2 3 1 2 3 1 2 3 1 2 3**

**CQ**


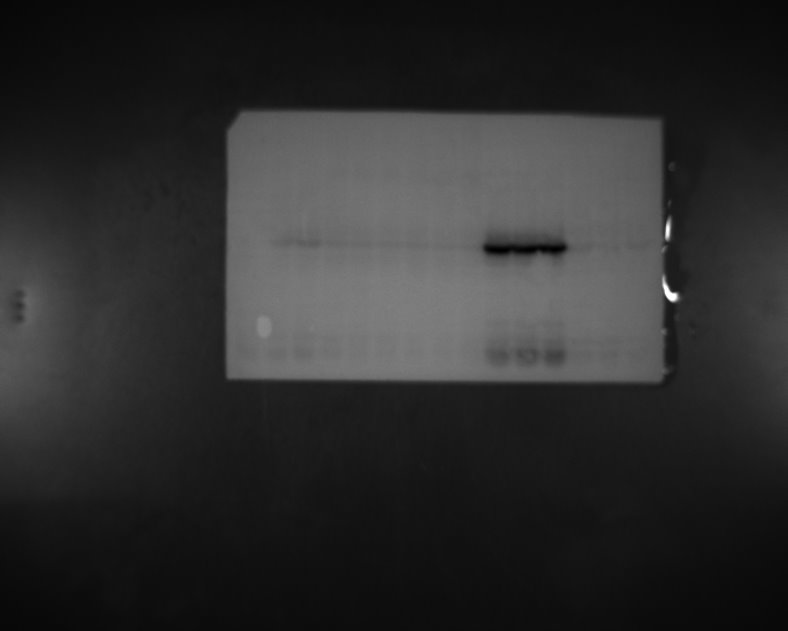


**CT 3-DAY 6-DAY 9-DAY 12-DAY**

**1 2 3 1 2 3 1 2 3 1 2 3 1 2 3**

**The blots of CD36 in MAC-T cells.**


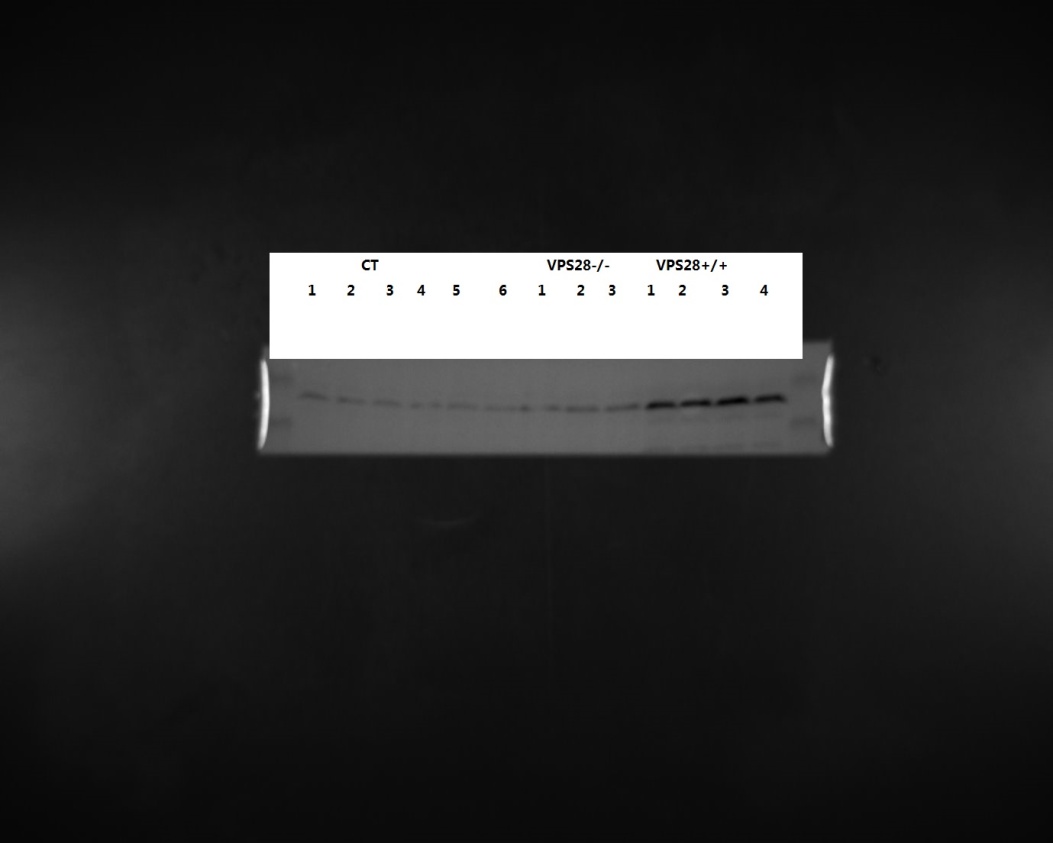

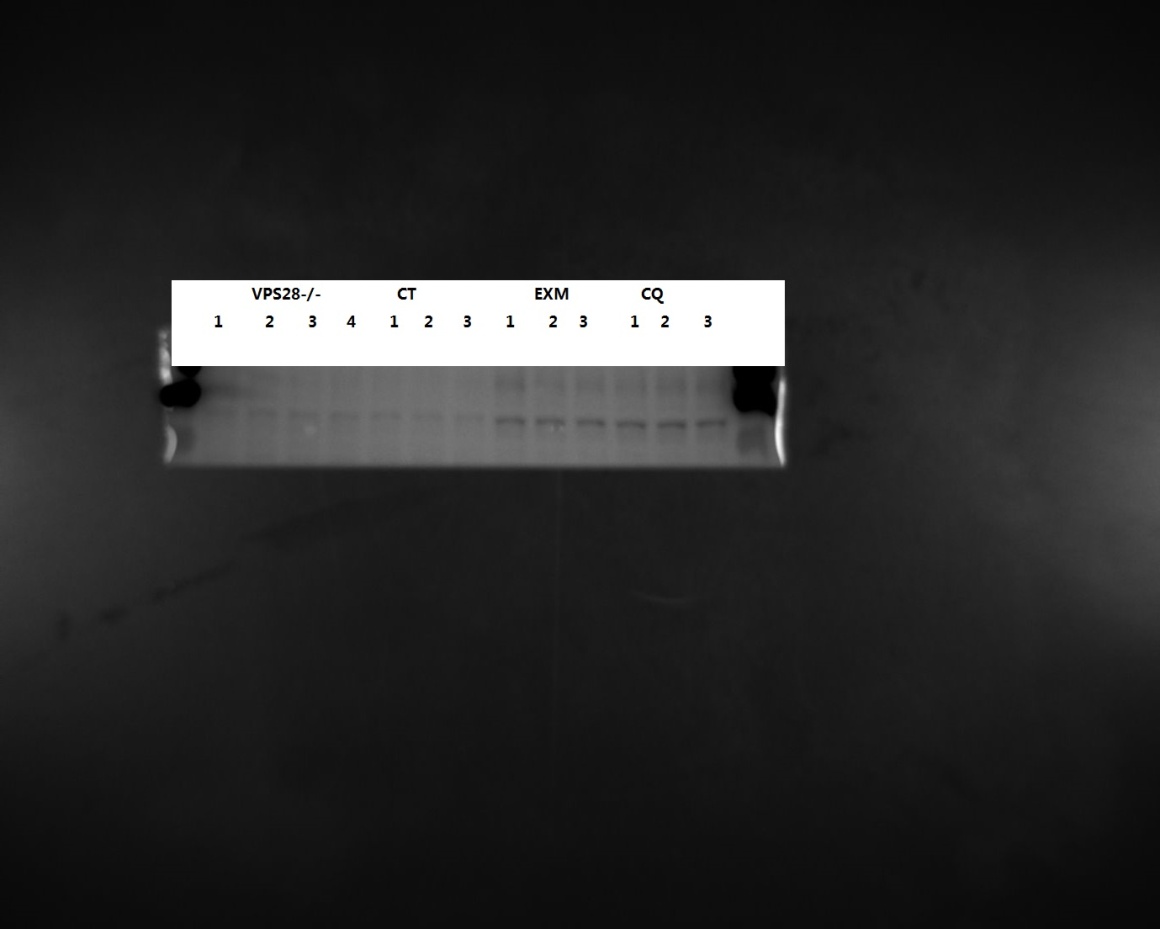


**The blots of CD36 in mice.**

**VPS28-/-**


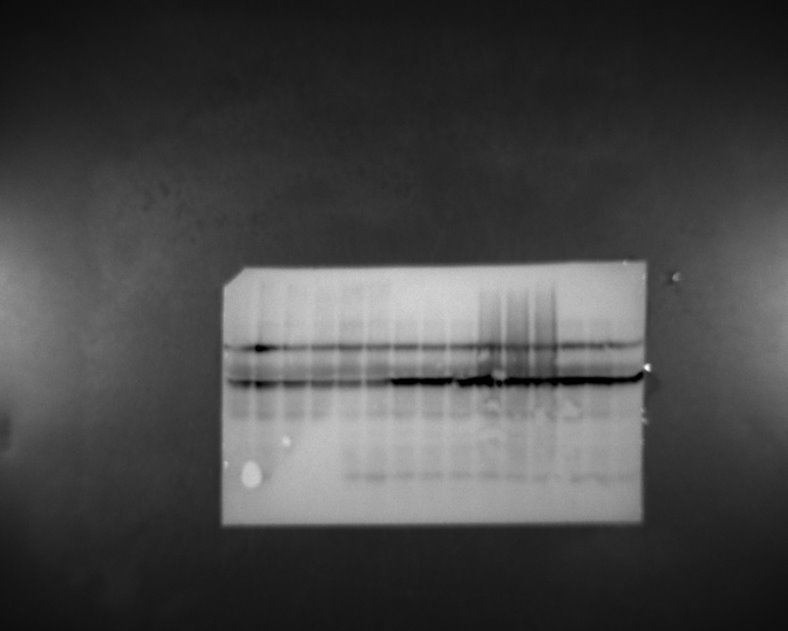


**CT 3-DAY 6-DAY 9-DAY 12-DAY**

**1 2 3 1 2 3 1 2 3 1 2 3 1 2 3**

**EXM**


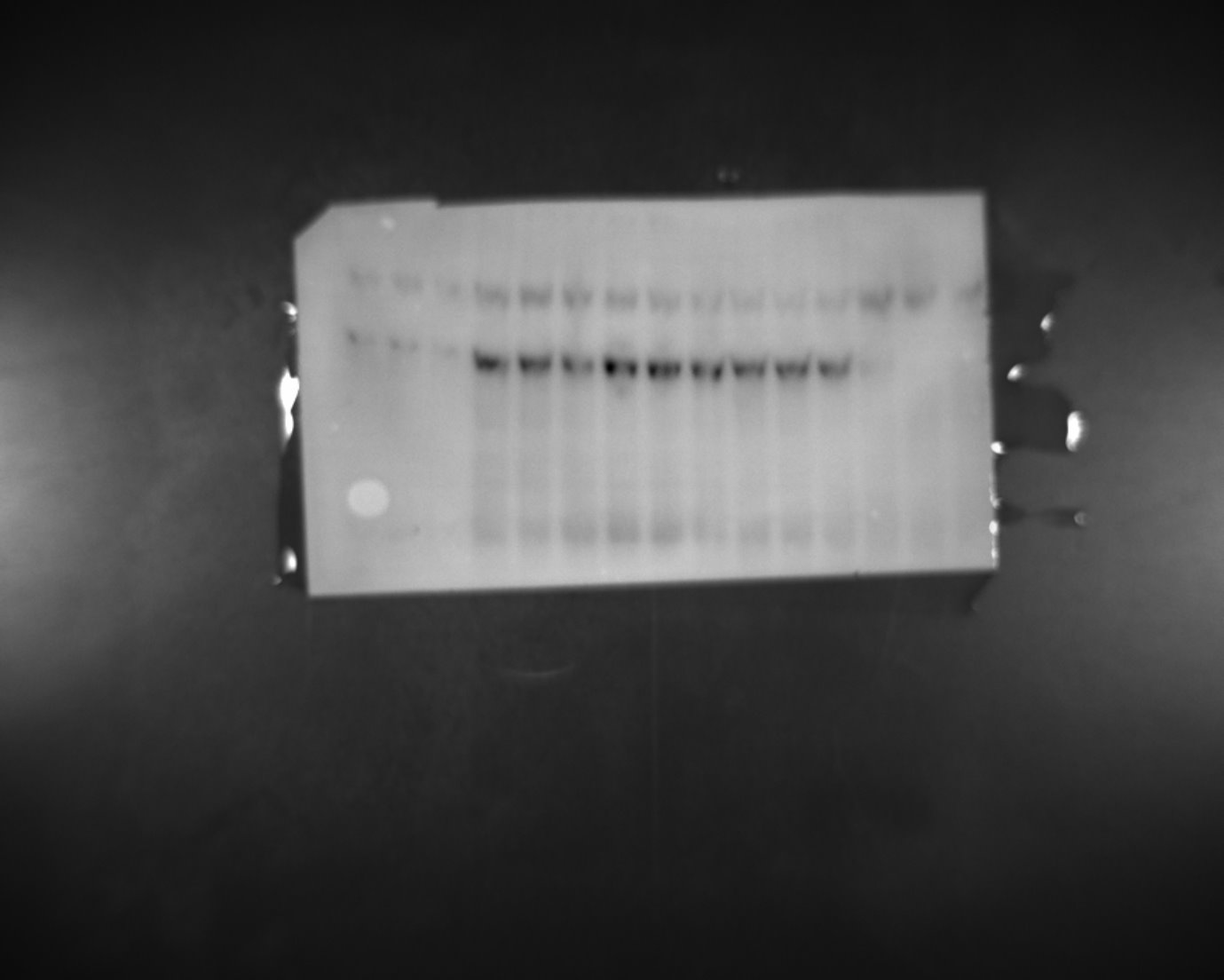


**CT 3-DAY 6-DAY 9-DAY 12-DAY**

**1 2 3 1 2 3 1 2 3 1 2 3 1 2 3**

**CQ**


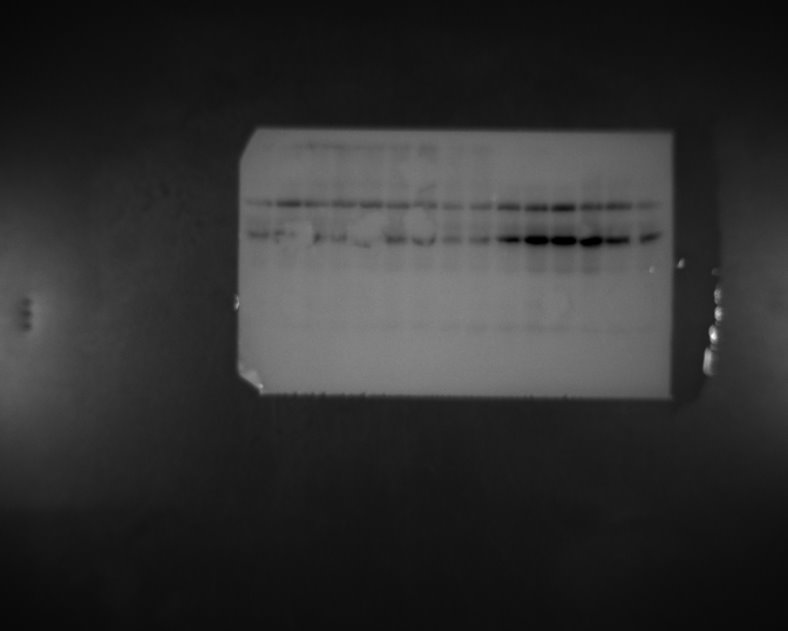


**CT 3-DAY 6-DAY 9-DAY 12-DAY**

**1 2 3 1 2 3 1 2 3 1 2 3 1 2 3**

**The blots of ADFP in MAC-T cells.**


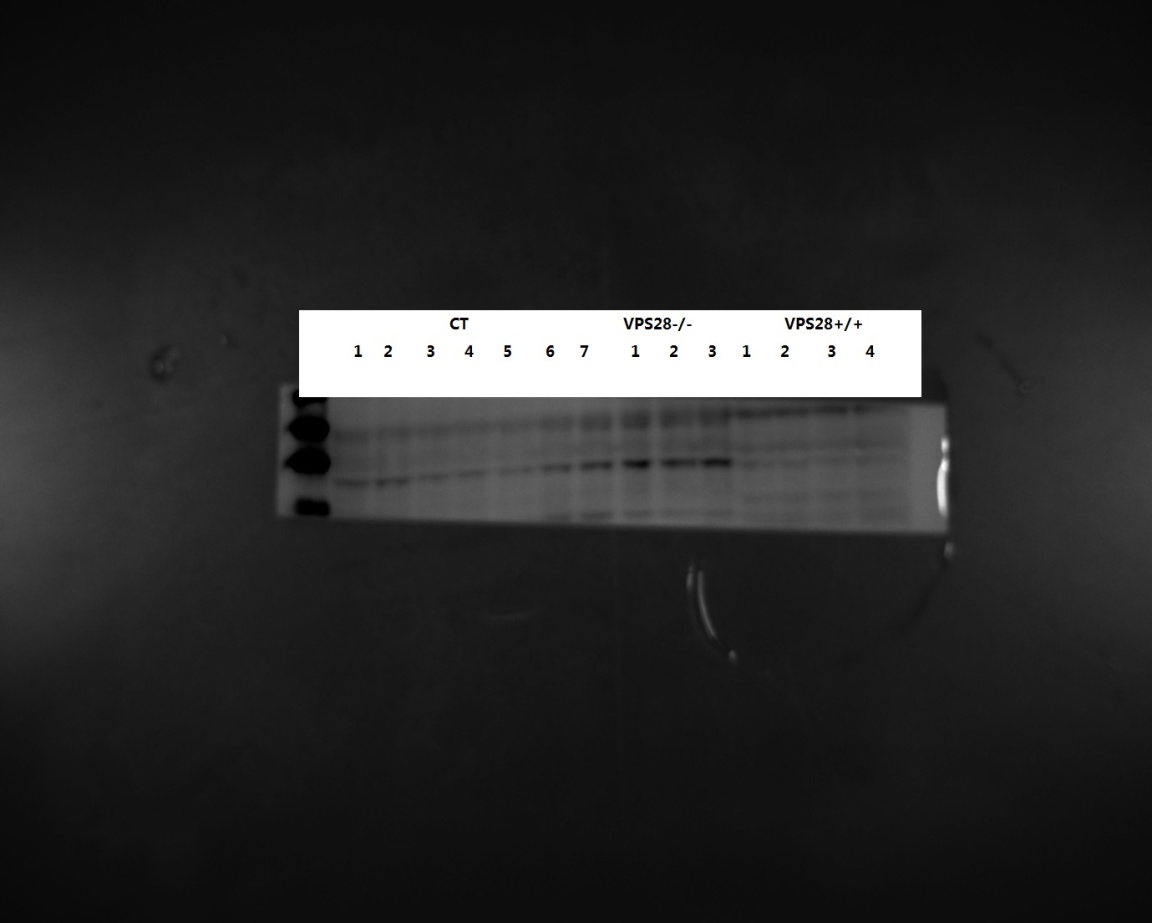

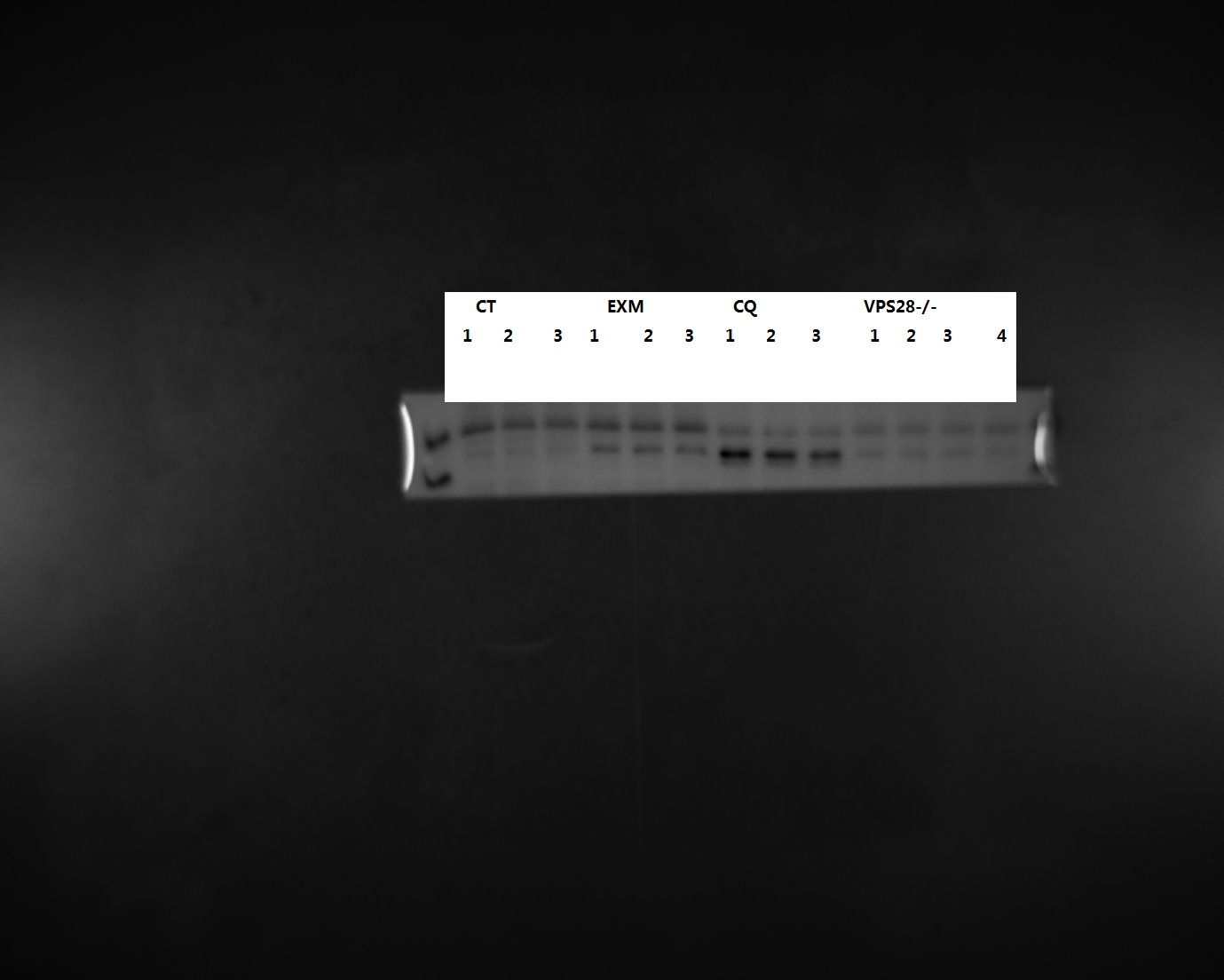


**The blots of ADFP in mice.**

**VPS28-/-**


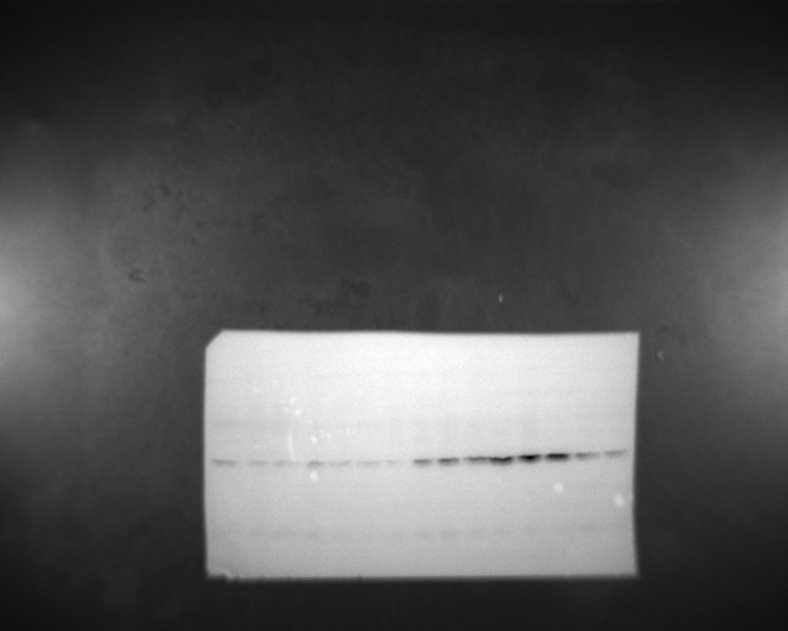


**CT 3-DAY 6-DAY 9-DAY 12-DAY**

**1 2 3 1 2 3 1 2 3 1 2 3 1 2 3**

**EXM**


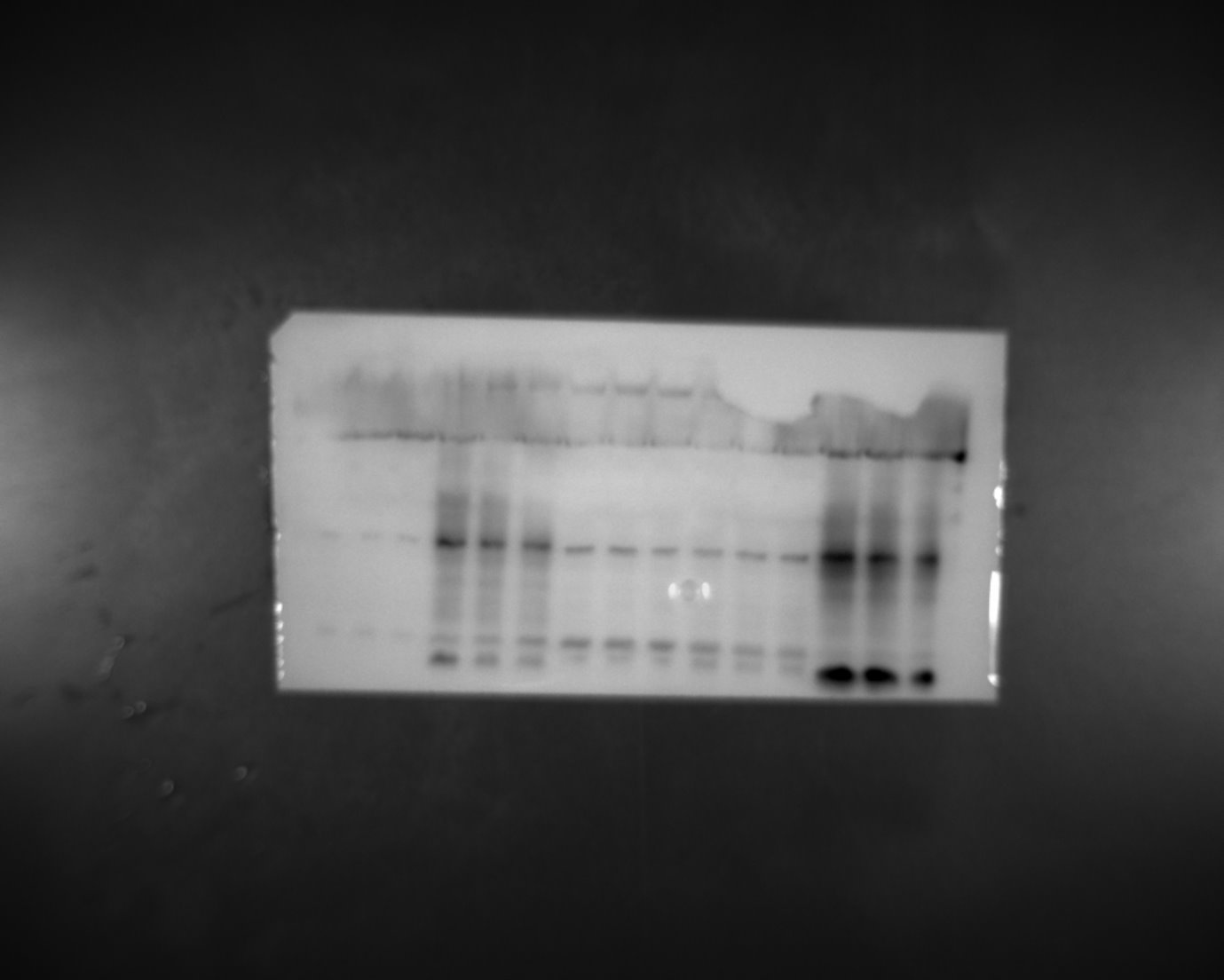


**CT 3-DAY 6-DAY 9-DAY 12-DAY**

**1 2 3 1 2 3 1 2 3 1 2 3 1 2 3**

**CQ**


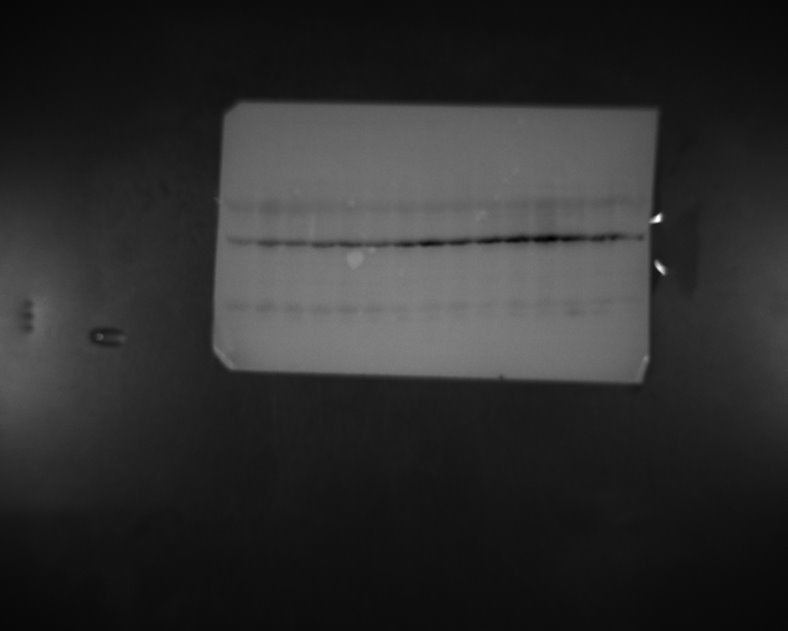


**CT 3-DAY 6-DAY 9-DAY 12-DAY**

**1 2 3 1 2 3 1 2 3 1 2 3 1 2 3**

**The blots of β-actin in MAC-T cells.**


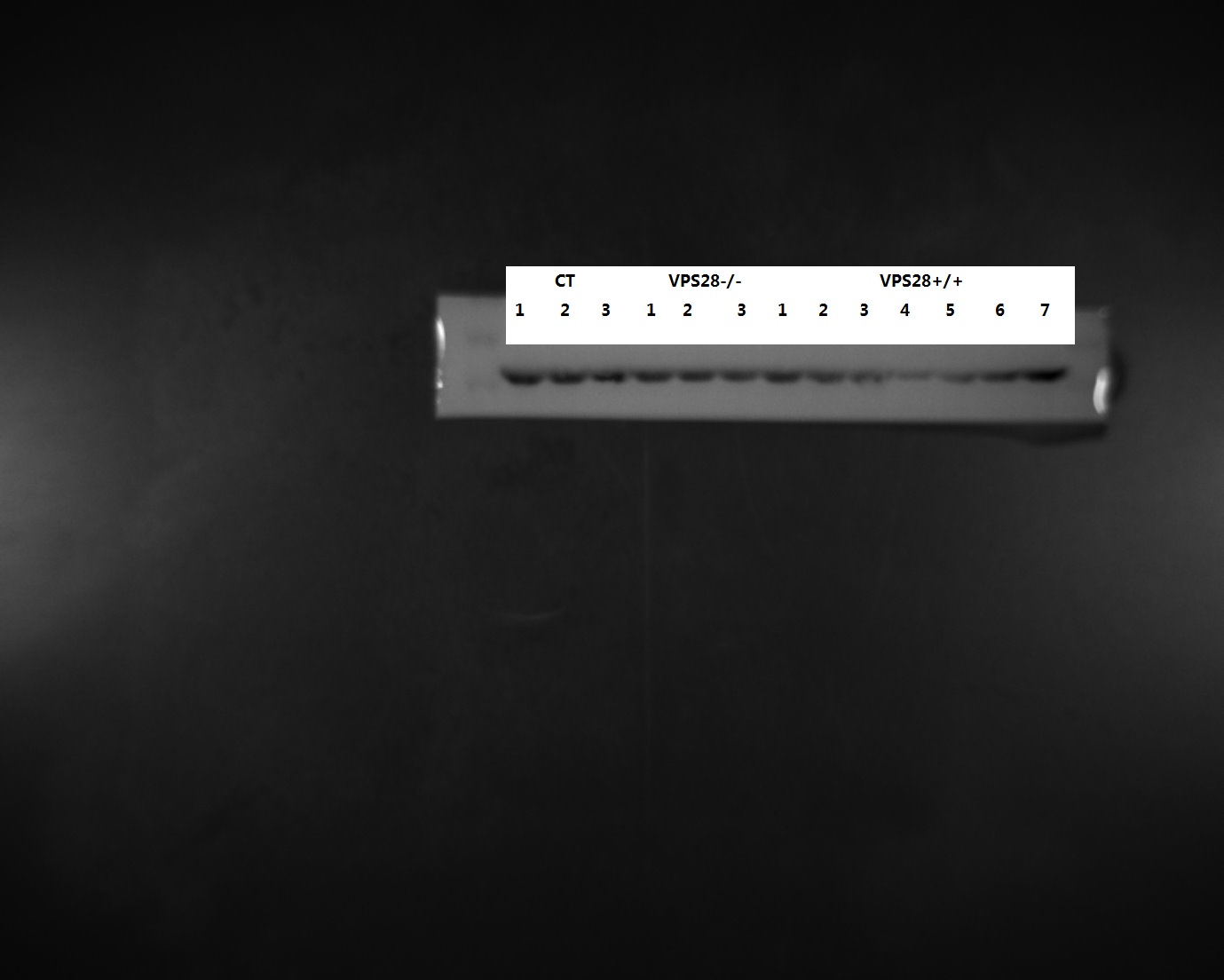


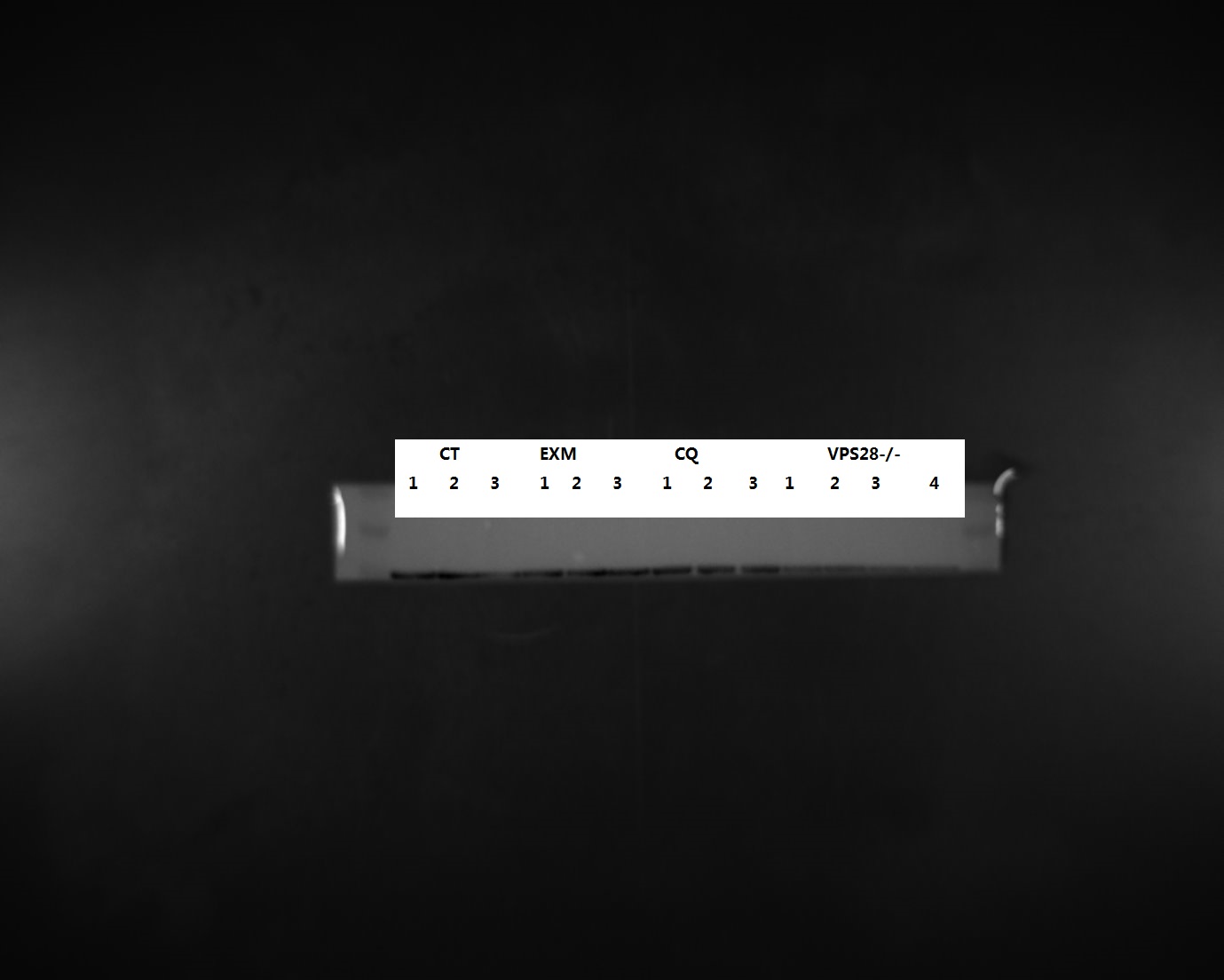


**The blots of β-actin in mice.**

**VPS28-/-**

**
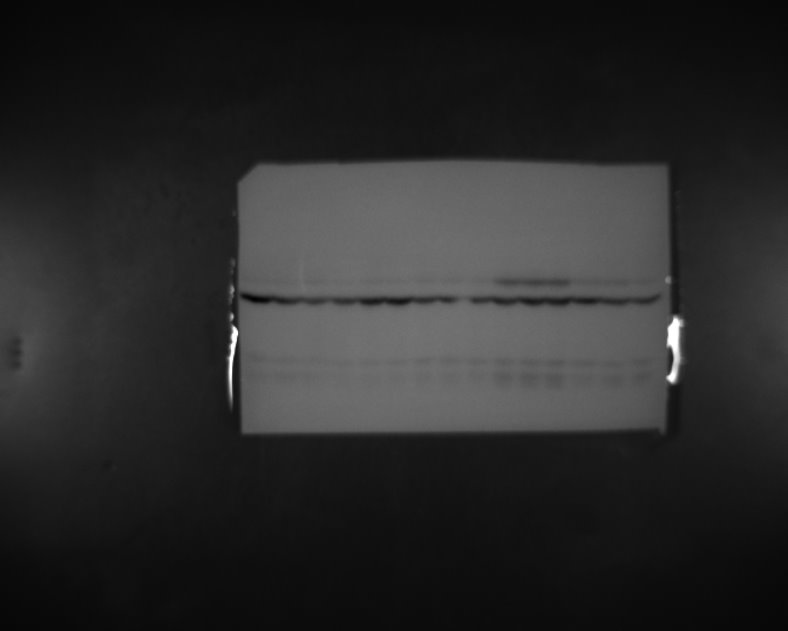
**

**CT 3-DAY 6-DAY 9-DAY 12-DAY**

**1 2 3 1 2 3 1 2 3 1 2 3 1 2 3**

**EXM**

**
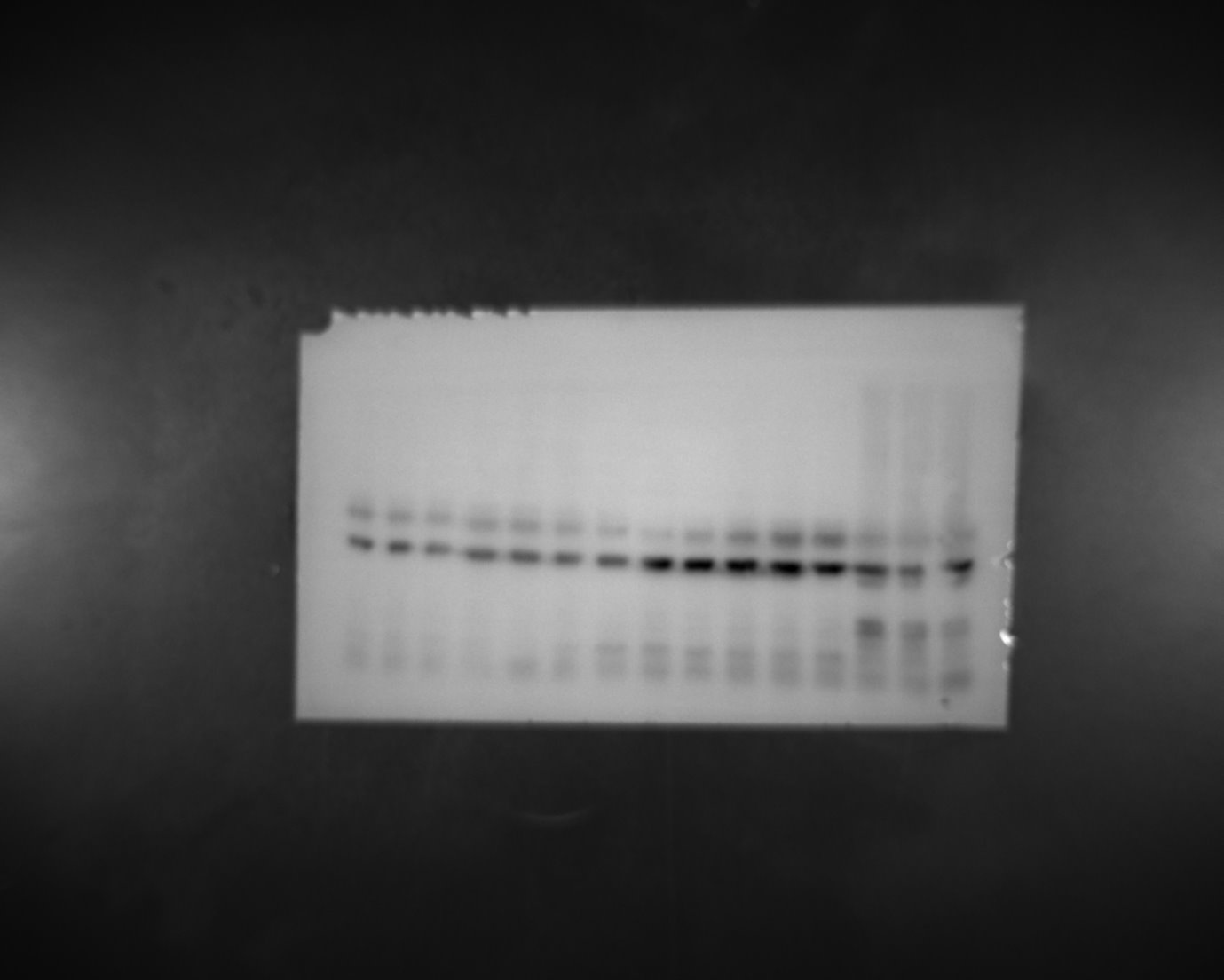
**

**CT 3-DAY 6-DAY 9-DAY 12-DAY**

**1 2 3 1 2 3 1 2 3 1 2 3 1 2 3**

**CQ**

**
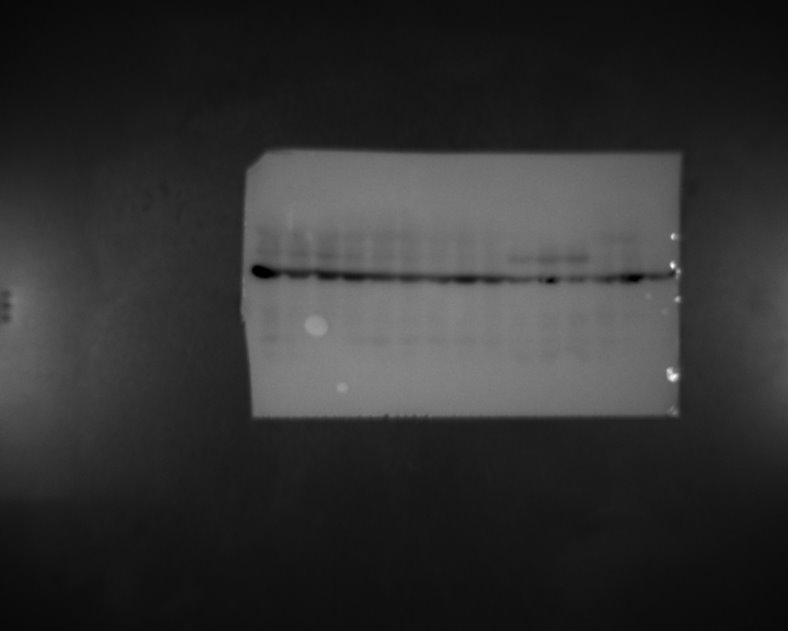
**

**CT 3-DAY 6-DAY 9-DAY 12-DAY**

**1 2 3 1 2 3 1 2 3 1 2 3 1 2 3**
